# Metabolic Remodeling during Nitrogen Fixation in Zymomonas mobilis

**DOI:** 10.1128/mSystems.00987-21

**Published:** 2021-11-16

**Authors:** Julia I. Martien, Edna A. Trujillo, Tyler B. Jacobson, Mehmet Tatli, Alexander S. Hebert, David M. Stevenson, Joshua J. Coon, Daniel Amador-Noguez

**Affiliations:** a DOE Great Lakes Bioenergy Research Center, University of Wisconsin–Madison, Madison, Wisconsin, USA; b Department of Bacteriology, University of Wisconsin–Madison, Madison, Wisconsin, USA; c Department of Chemistry, University of Wisconsin–Madison, Madison, Wisconsin, USA; d National Center for Quantitative Biology of Complex Systems, University of Wisconsin–Madison, Madison, Wisconsin, USA; e Department of Biomolecular Chemistry, University of Wisconsin–Madison, Madison, Wisconsin, USA; f Morgridge Institute for Research, Madison, Wisconsin, USA; CSIRO

**Keywords:** MEP pathway, *Zymomonas mobilis*, biofuels, isoprenoids, metabolomics, nitrogen fixation, nitrogen metabolism, proteomics, systems biology, thermodynamics

## Abstract

Zymomonas mobilis is an ethanologenic bacterium currently being developed for production of advanced biofuels. Recent studies have shown that Z. mobilis can fix dinitrogen gas (N_2_) as a sole nitrogen source. During N_2_ fixation, Z. mobilis exhibits increased biomass-specific rates of ethanol production. In order to better understand the physiology of Z. mobilis during N_2_ fixation and during changes in ammonium (NH_4_^+^) availability, we performed liquid chromatography-mass spectrometry (LC-MS)-based targeted metabolomics and shotgun proteomics under three regimes of nitrogen availability: continuous N_2_ fixation, gradual NH_4_^+^ depletion, and acute NH_4_^+^ addition to N_2_-fixing cells. We report dynamic changes in abundance of proteins and metabolites related to nitrogen fixation, motility, ammonium assimilation, amino acid biosynthesis, nucleotide biosynthesis, isoprenoid biosynthesis, and Entner-Doudoroff (ED) glycolysis, providing insight into the regulatory mechanisms that control these processes in Z. mobilis. Our analysis identified potential physiological mechanisms that may contribute to increased specific ethanol production during N_2_ fixation, including decreased activity of biosynthetic pathways, increased protein abundance of alcohol dehydrogenase (ADHI), and increased thermodynamic favorability of the ED pathway. Of particular relevance to advanced biofuel production, we found that intermediates in the methylerythritol phosphate (MEP) pathway for isoprenoid biosynthesis were depleted during N_2_ fixation, coinciding with decreased protein abundance of deoxyxylulose 5-phosphate synthase (DXS), the first enzyme in the pathway. This implies that DXS protein abundance serves as a native control point in regulating MEP pathway activity in Z. mobilis. The results of this study will inform metabolic engineering to further develop Z. mobilis as a platform organism for biofuel production.

**IMPORTANCE** Biofuels and bioproducts have the potential to serve as environmentally sustainable replacements for petroleum-derived fuels and commodity molecules. Advanced fuels such as higher alcohols and isoprenoids are more suitable gasoline replacements than bioethanol. Developing microbial systems to generate advanced biofuels requires metabolic engineering to reroute carbon away from ethanol and other native products and toward desired pathways, such as the MEP pathway for isoprenoid biosynthesis. However, rational engineering of microbial metabolism relies on understanding metabolic control points, in terms of both enzyme activity and thermodynamic favorability. In Z. mobilis, the factors that control glycolytic rates, ethanol production, and isoprenoid production are still not fully understood. In this study, we performed metabolomic, proteomic, and thermodynamic analysis of Z. mobilis during N_2_ fixation. This analysis identified key changes in metabolite levels, enzyme abundance, and glycolytic thermodynamic favorability that occurred during changes in NH_4_^+^ availability, helping to inform future efforts in metabolic engineering.

## INTRODUCTION

Zymomonas mobilis has long been recognized as a promising platform organism for biofuel production (1–3). A combination of high glucose tolerance, rapid glucose consumption, and high ethanol yield (over 90% theoretical maximum) make Z. mobilis comparable or even superior to Saccharomyces cerevisiae in its native ability to produce bioethanol (4–6). Ongoing efforts in metabolic engineering aim to further increase the utility of Z. mobilis as a biofuel producer by expanding its substrate utilization, improving its stress tolerance, and diversifying its product profiles to include more valuable products such as higher alcohols and isoprenoids (7–16, 114, 115).

Recently, it was demonstrated that Z. mobilis is capable of fixing dinitrogen gas (N_2_) as a sole nitrogen source (17). The ability to utilize N_2_ gas rather than bioavailable nitrogen supplements offers a clear advantage for lignocellulosic biofuel production in terms of both economic viability and environmental sustainability (17, 18). However, little is known about the physiology of Z. mobilis during N_2_ fixation. Previous studies have shown that during N_2_ fixation, Z. mobilis exhibits a lower growth rate and lower growth yield but a higher biomass-specific ethanol production rate, higher specific glucose consumption rate, and equivalent or slightly higher ethanol yield (17, 19, 20). This is a striking observation considering the already highly catabolic metabolism employed by Z. mobilis during replete ammonium (NH_4_^+^) availability (21). Increased glucose uptake and ethanol production imply major metabolic remodeling. However, the underlying physiological changes that occur during N_2_ fixation, including changes in protein expression and intracellular metabolite abundance, are currently unknown. N_2_ fixation therefore provides a unique system in which to examine native metabolic regulation in Z. mobilis and identify metabolic engineering strategies to maximize production of target molecules.

In this study, we measured relative protein abundance and relative abundance of intracellular metabolites using liquid chromatography coupled to mass spectrometry (LC-MS) (22, 23). LC-MS-based targeted metabolomics and shotgun proteomics were performed during continuous N_2_ fixation and during dynamic changes in NH_4_^+^ availability. This analysis expands the current understanding of Z. mobilis physiology and provides new information regarding the native regulation of biofuel-producing pathways.

## RESULTS AND DISCUSSION

### Experimental design and nitrogen availability regimes.

We quantified relative metabolite and protein abundance under N_2_-fixing conditions in comparison to NH_4_^+^-replete conditions and during transitions between these two growth conditions. Z. mobilis (ATCC 31821) was grown anaerobically using glucose as the sole carbon source (see Materials and Methods) (24). For conditions of replete NH_4_^+^ availability, 15 mM NH_4_^+^ was provided. For N_2_-fixing conditions, no NH_4_^+^ was added to the medium and the only available nitrogen source was N_2_ gas (>90% N_2_ in the anaerobic chamber).

**FIG 1:**
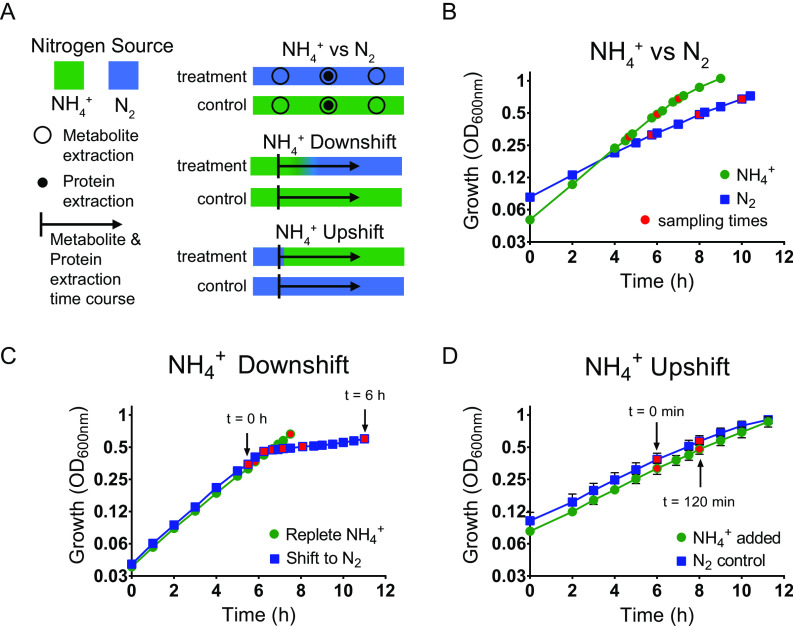
(A) Schematic of experimental design. See Materials and Methods for more details. (B to D) Representative growth of Z. mobilis cultures measured by optical density at 600 nm. (B) Continuous N_2_-fixing conditions (blue squares) compared to continuous NH_4_^+^-replete conditions (15 mM NH_4_^+^) (green circles). Times of metabolite extractions are shown as red symbols. Protein extractions were conducted at an OD_600_ of ∼0.5. Data points are the average of 3 biological replicates. Error bars showing standard deviation are smaller than the height of symbols. (C) Replete (15 mM) NH_4_^+^ controls (green circles) compared to NH_4_^+^ downshift (starting with <5 mM NH_4_^+^) (blue squares). Arrows indicate start and end of time course. Times of metabolite and protein extraction are shown as red symbols. Data points are the average of 3 biological replicates for NH_4_^+^ downshift and 2 biological replicates for controls. Error bars showing standard deviation are smaller than the height of the symbols. (D) Continuous N_2_-fixing controls (blue squares) compared to NH_4_^+^ upshift (15 mM NH_4_Cl was added to N_2_-fixing cultures at *t* = 0) (green circles). Arrows and red symbols indicate metabolite and protein extractions taken at start and end of time course. See Materials and Methods for a full list of time points within this time course. Data points are the average of 3 biological replicates for NH_4_^+^ upshift and 2 biological replicates for controls. Error bars show the standard deviation.

We examined three separate regimes of nitrogen availability (Fig. 1A). (i) Continuous N_2_-fixing conditions were compared to continuous NH_4_^+^-replete conditions; doubling times were approximately 3 h during N_2_ fixation and 2 h under NH_4_^+^-replete conditions (Fig. 1B). Samples were taken for targeted metabolomics at early, mid-, and late exponential phase and for shotgun proteomics at mid-exponential phase. (ii) NH_4_^+^ downshift; Z. mobilis was grown in medium containing limited NH_4_^+^ (<2 mM) such that growth stalled at mid-exponential phase. During the dynamic shift to N_2_ fixation, a 6-h metabolomics and proteomics time course was conducted. Samples were collected for the initial (*t* = 0) time point during early exponential growth before a decrease in growth rate was observed, i.e., when the doubling time was still ∼2 h (Fig. 1C). Samples were also taken from NH_4_^+^-replete controls which grew with a doubling time of 2 h (Fig. 1C). (iii) NH_4_^+^ upshift; Z. mobilis was grown under N_2_-fixing conditions until early exponential phase, at which point NH_4_Cl was added to the medium at a final concentration of 15 mM. During NH_4_^+^ upshift, a 2-h metabolomics and proteomics time course was conducted. Samples were collected for the initial (*t* = 0) time point immediately before addition of NH_4_Cl. Samples were also taken from N_2_-fixing controls. Both conditions grew with a consistent 3-h doubling time for the duration of the 2-h time course (Fig. 1D).

### Metabolome analysis reveals global alterations in intracellular metabolite levels in response to changes in nitrogen availability.

**FIG 2:**
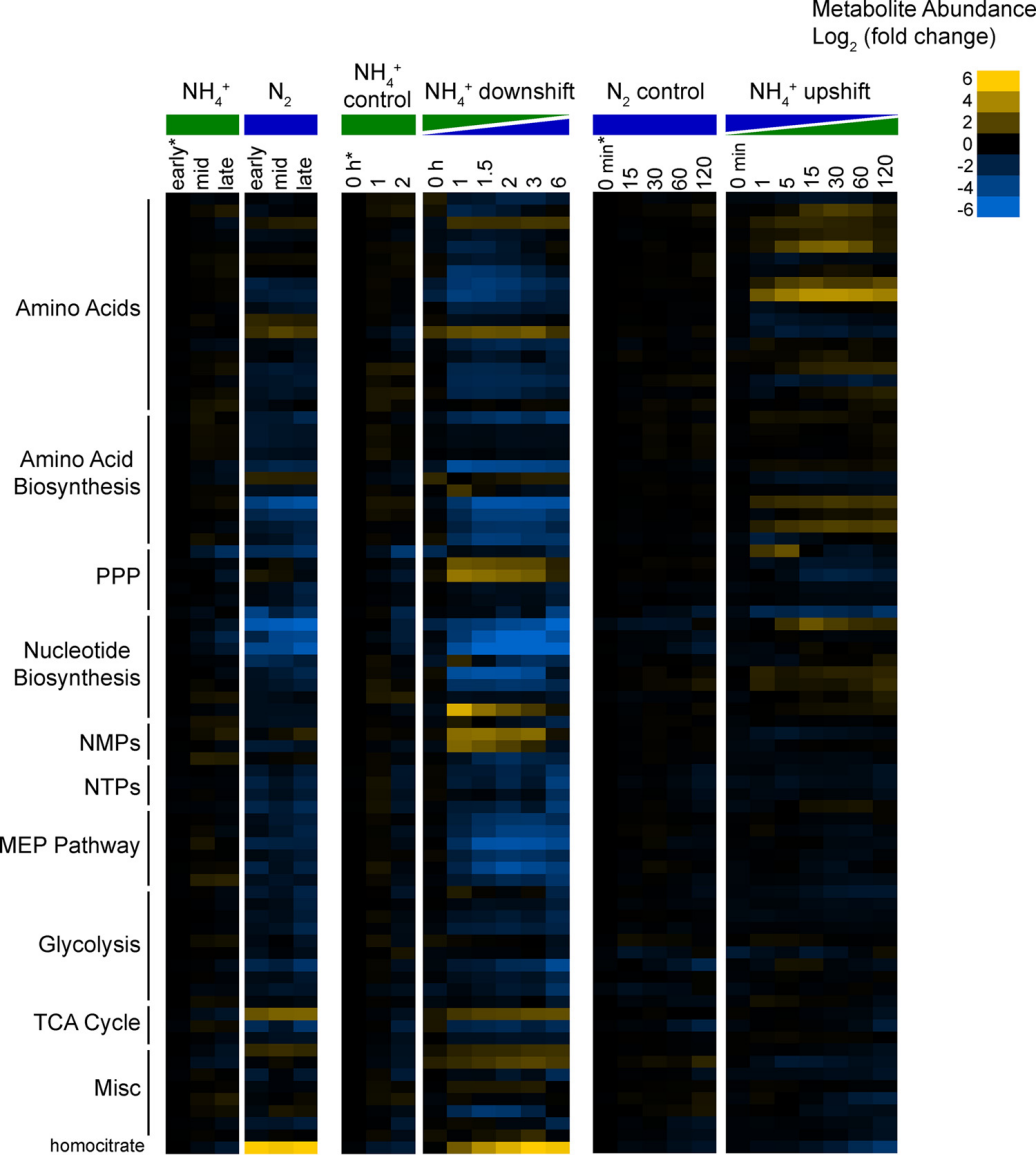
Relative intracellular metabolite abundance of the 79 metabolites that were differentially abundant (fold change of ≥1.5 and FDR-adjusted *P* value of ≤0.05) during at least one of the three conditions of nitrogen availability; from left to right, continuous NH_4_^+^-replete conditions versus continuous N_2_-fixing conditions, NH_4_^+^ downshift (shift to N_2_-fixing conditions), and NH_4_^+^ upshift (from N_2_-fixing conditions), respectively. Rows are a single metabolite across all conditions, and columns are individual metabolomics samples, taken at the indicated times and conditions. Log_2_ fold changes are relative to the first time point in the control condition for each experiment (indicated by an asterisk). Values are the averages of 2 biological replicates for time course controls, 3 biological replicates for time course treatments, and 5 biological replicates for both continuous conditions. Yellow corresponds to increased intracellular metabolite abundance compared to the control, and blue indicates depletion. Metabolites were manually arranged based on the biosynthetic pathway.

Metabolomic analysis using LC-MS produced relative intracellular abundance for 99 unique metabolites spanning central carbon metabolism (see Table S1 in the supplemental material). These included intermediates of Entner-Doudoroff (ED) glycolysis, the pentose phosphate pathway (PPP), the tricarboxylic acid (TCA) cycle, amino acid biosynthesis, nucleotide biosynthesis, isoprenoid biosynthesis, and peptidoglycan biosynthesis. Of the 99 detected metabolites, 79 were differentially abundant (fold change [FC] of  >1.5 and false discovery rate [FDR]-adjusted *P* value of <0.05) during at least one of the three conditions of nitrogen availability (Fig. 2). In general, amino acids and intermediates in amino acid biosynthesis were depleted during N_2_ fixation and either increased or remained constant in response to NH_4_^+^ upshift. One notable exception was arginine, which increased during N_2_ fixation and decreased after NH_4_^+^ addition. Intermediates of *de novo* nucleotide biosynthesis were severely depleted (>30-fold decrease) under N_2_-fixing conditions. Nucleotide triphosphates (NTPs) were also less abundant during N_2_ fixation, although to a lesser extent (∼4-fold decrease). Conversely, intracellular concentrations of nucleosides and nucleotide monophosphates (NMPs) increased during the shift to N_2_ fixation. There were dynamic changes in the PPP during shifts in NH_4_^+^ availability, which are likely linked to nucleotide biosynthesis. We observed depletion of intermediates in both the ED glycolytic pathway and the methylerythritol phosphate (MEP) pathway for isoprenoid biosynthesis during N_2_ fixation. Overall, intracellular metabolite levels changed dramatically during the shift to N_2_ fixation but remained much more consistent during NH_4_^+^ upshift, where the largest changes corresponded to increased amino acid abundance, particularly glutamine.

10.1128/mSystems.00987-21.7 TABLE S1 Relative intracellular metabolite abundance of all 99 metabolites that were quantified by targeted LC-MS-based metabolomics. Download Table S1, XLSX file, 0.06 MB. Copyright © 2021 Martien et al. 2021 Martien et al. https://creativecommons.org/licenses/by/4.0/ This content is distributed under the terms of the Creative Commons Attribution 4.0 International license.

### Proteome remodeling in response to changes in nitrogen availability.

**FIG 3:**
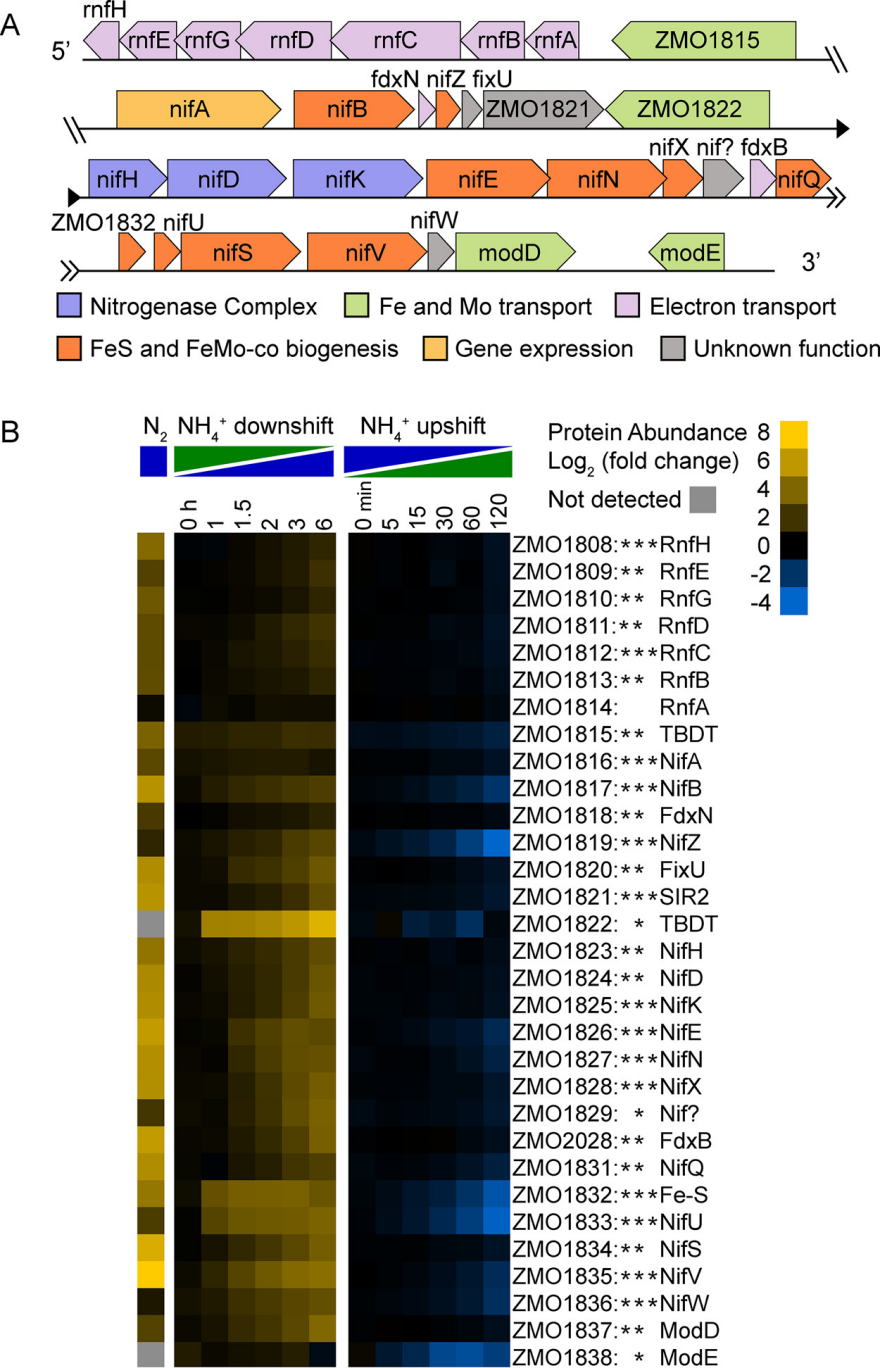
(A) Schematic of the *nif* gene cluster in Z. mobilis. For each gene, the arrow length is representative of the approximate length of the coding region and the arrow direction corresponds to the direction of transcription. Colors were assigned based on gene function. (B) Relative protein abundance of gene products from the *nif* cluster. Log_2_ fold changes are relative to the first time point in the control condition (not shown). Values are the averages of at least 3 biological replicates. Asterisks indicate statistical significance (FC ≥ 1.5, FDR ≤ 0.05) for NH_4_^+^ versus N_2_, NH_4_^+^ downshift, and NH_4_^+^ upshift experiments, respectively, from left to right. For example, RnfH protein abundance changed significantly for all three conditions, but changes in RnfE abundance were only significant for NH_4_^+^ versus N_2_ and NH_4_^+^ downshift conditions. A gray tile indicates that protein was not detected under that condition. Proteins were manually arranged based on genomic location within the *nif* cluster.

Proteomics analysis during NH_4_^+^ downshift and NH_4_^+^ upshift produced relative protein abundance for 1,693 unique proteins (90% coverage of protein coding genes) (22, 25, 26). The comparison between continuous N_2_ and NH_4_^+^ growth conditions was performed separately and yielded 1,429 proteins (75% coverage). Of the proteins detected, 615 changed significantly during at least one of the three conditions of N_2_ availability (FC of >1.5 and FDR-adjusted *P* value of <0.05) (Table S2). We identified 296 proteins that were affected during continuous N_2_ fixation relative to continuous replete NH_4_^+^, 467 proteins that were differentially abundant during the shift to N_2_ fixation, and only 33 proteins that responded to the NH_4_^+^ upshift. We performed an overrepresentation analysis of gene ontology (GO) terms associated with the set of proteins found to be differentially abundant under each of the three conditions (Table S3) (27). All three conditions yielded “nitrogen fixation,” “nitrogenase activity,” and “iron-sulfur cluster binding” as terms that were enriched among affected proteins. Proteins that were differentially abundant during the N_2_ versus NH_4_^+^ conditions were also enriched for “regulation of nitrogen utilization,” “cellular iron ion homeostasis,” “cysteine desulfurase activity,” and “arginine biosynthetic process via ornithine” among other terms. Proteins that changed in abundance during NH_4_^+^ downshift were also enriched for GO terms such as “siderophore uptake transmembrane transporter activity,” “bacterial-type flagellum,” “oxidoreductase activity,” and “response to oxidative stress.” Products of the nitrogen fixation (*nif*) gene cluster were consistently among the most affected proteins under all three conditions of NH_4_^+^ availability (Fig. 3). During the transition to N_2_ fixation, proteins involved in metal transport, electron transport, and oxidative stress increased in abundance, whereas proteins involved in translation and motility decreased in abundance (Fig. S1 and S2). Our integrated metabolomics and proteomics analysis in Z. mobilis also revealed widespread and dynamic remodeling of metabolism in response to changes in nitrogen availability. In the following sections, we summarize a subset of the most significant alterations.

10.1128/mSystems.00987-21.1 FIG S1 Relative protein abundance of the top 100 differentially abundant (fold change of >1.5 and FDR-adjusted *P* value of <0.05) proteins, not including products of the *nif* gene cluster. Proteins shown were differentially abundant during at least one of the three conditions of nitrogen availability: from left to right, continuous N_2_-fixing conditions (compared to NH_4_^+^-replete controls), gradual NH_4_^+^ downshift (compared to NH_4_^+^-replete controls), and acute NH_4_^+^ upshift (compared to N_2_-fixing controls). The full list of differentially abundant proteins can be found in Table S2. Rows are a single protein across all conditions, and columns are individual proteomics samples, taken at the indicated times and conditions. Log_2_ fold changes are relative to the first time point in the control condition for each experiment (indicated with an asterisk). Values are the averages of 2 biological replicates for time course controls, 3 biological replicates for time course treatments, and 5 biological replicates for continuous conditions. Yellow corresponds to increased protein abundance compared to the control, and blue indicates depletion. A gray tile indicates that the protein was not identified in that sample. Proteins were manually arranged based on biological function. Download FIG S1, EPS file, 1.3 MB. Copyright © 2021 Martien et al. 2021 Martien et al. https://creativecommons.org/licenses/by/4.0/ This content is distributed under the terms of the Creative Commons Attribution 4.0 International license.

10.1128/mSystems.00987-21.2 FIG S2 Relative abundance of protein products from the flagellar gene cluster (ZMO0602-ZMO0652) and the chemotaxis gene cluster (ZMO0078-ZMO0085) during three conditions of nitrogen availability: from left to right, continuous NH_4_^+^-replete conditions versus continuous N_2_-fixing conditions, gradual NH_4_^+^ downshift, and acute NH_4_^+^ upshift, respectively. Rows are a single protein across all conditions, and columns are individual proteomics samples, taken at the indicated times and conditions. Log_2_ fold change values are relative to the first time point in the control condition (not shown). Values are the averages of at least 3 biological replicates. Yellow corresponds to increased protein abundance compared to the control, and blue indicates decreased protein abundance. Asterisks indicate statistical significance (FC ≥ 1.5, FDR ≤ 0.05) for NH_4_^+^ versus N_2_, NH_4_^+^ downshift, and NH_4_^+^ upshift conditions, respectively, from left to right. A gray tile indicates that the protein was not identified in that sample. Proteins were manually arranged based on genomic location within the two gene clusters. Download FIG S2, EPS file, 1.1 MB. Copyright © 2021 Martien et al. 2021 Martien et al. https://creativecommons.org/licenses/by/4.0/ This content is distributed under the terms of the Creative Commons Attribution 4.0 International license.

10.1128/mSystems.00987-21.8 TABLE S2 Relative protein abundance of all proteins that were differentially abundant during at least one of the three conditions of nitrogen availability and complete list of relative protein abundance for all detected proteins. Download Table S2, XLSX file, 0.6 MB. Copyright © 2021 Martien et al. 2021 Martien et al. https://creativecommons.org/licenses/by/4.0/ This content is distributed under the terms of the Creative Commons Attribution 4.0 International license.

10.1128/mSystems.00987-21.9 TABLE S3 Results of gene ontology analysis. Download Table S3, XLSX file, 0.02 MB. Copyright © 2021 Martien et al. 2021 Martien et al. https://creativecommons.org/licenses/by/4.0/ This content is distributed under the terms of the Creative Commons Attribution 4.0 International license.

### Concerted increase in the abundance of nitrogenase and nitrogenase-supporting enzymes during N_2_-fixing conditions.

In Z. mobilis, as in other nitrogen-fixing bacteria, the genes required for N_2_ fixation are clustered within a 30-kb genomic region called the *nif* cluster (28, 29). The Z. mobilis *nif* cluster contains 31 genes: *nifABZHDKENXQUSVW*, *fdxN*, *fixU*, *fdxB*, *modD*, *modE*, the *rnfABCDGEH* operon, a gene (ZMO1832) encoding an iron-sulfur cluster assembly accessory protein, two genes (ZMO1815 and ZMO1822) encoding iron-associated TonB-dependent transporters (TBDT), an uncharacterized N_2_ fixation gene (ZMO1829), and a gene (ZMO1821) encoding a hypothetical protein with an SIR2-like domain (30) (Fig. 3A). The *nif* cluster codes for the three proteins that form the active nitrogenase complex: nitrogenase reductase (NifH), also called the Fe protein, and the α and β subunits of nitrogenase (NifD and NifK), also called the MoFe protein. Several genes in the *nif* cluster (e.g., *nifB*, *nifU nifE*, *nifN*, and *nifV*) are involved in the biogenesis of iron-sulfur clusters required for N_2_ fixation, including the [Fe_4_-S_4_] cluster cofactor of NifH, the [Fe_8_-S_7_] cluster (P-cluster) cofactor of NifDK, and the [Mo-Fe_7_-S_9_-C-homocitrate] molybdenum-iron cofactor (FeMo-co) at the active site of NifDK (31, 32). The *nif* gene cluster also contains the *rnf* operon, whose products form a membrane-bound complex that couples ion translocation across the inner membrane to the transfer of electrons from NADH to ferredoxin (e.g., FdxN or FdxB), which then donates electrons to the nitrogenase complex (33, 34). The *nif* cluster is regulated by the σ^54^-dependent transcription factor NifA, which is also encoded within the *nif* cluster (35–39).

**FIG 4:**
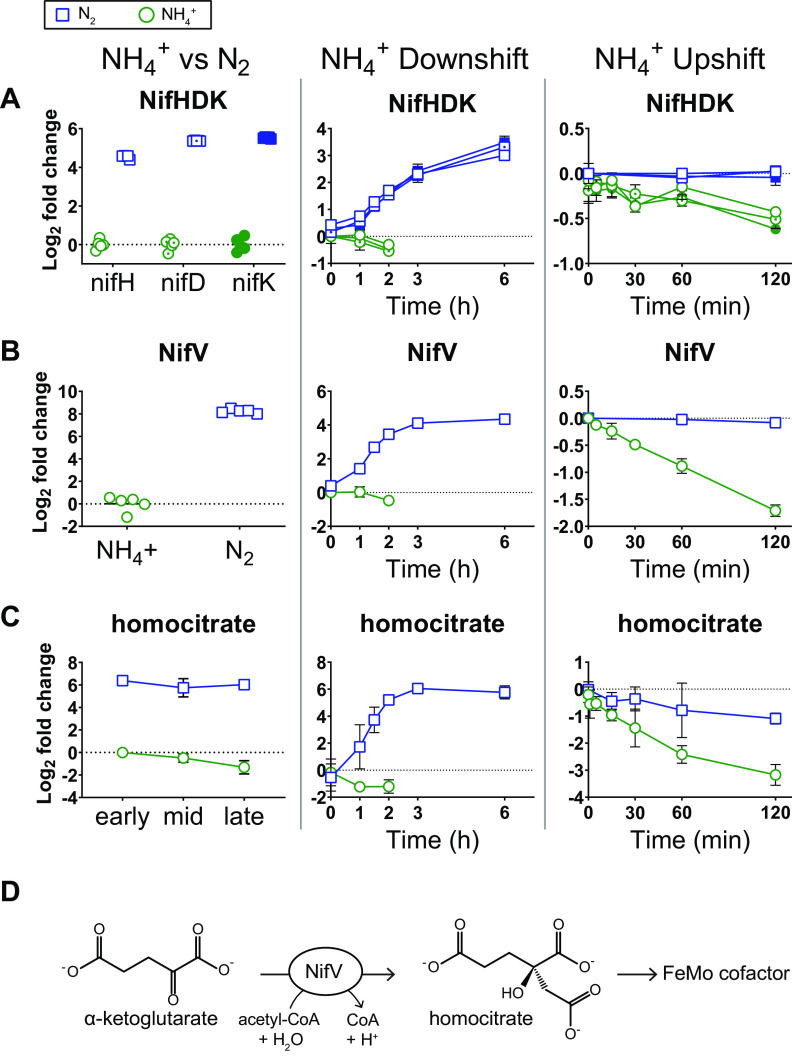
Relative abundance of proteins and metabolites related to nitrogenase function during three conditions of nitrogen availability. Blue squares indicate N_2_ fixation or transition to N_2_ fixation. Green circles indicate replete NH_4_^+^ or NH_4_^+^ upshift. Log_2_ fold change values are relative to the first time point in the control condition. For NH_4_^+^ versus N_2_ protein abundance, individual data points are shown (5 biological replicates per condition). For all other graphs, values are the average of at least 3 biological replicates for the treatment condition and at least 2 biological replicates for controls. Error bars show standard deviations (SD). (A) Protein abundance of nitrogenase structural proteins, including Fe protein (NifH) (open symbols), α subunit of MoFe protein (NifD) (open symbols with dots), and β subunit of MoFe protein (NifK) (closed symbols). (B) Protein abundance of homocitrate synthase (NifV). (C) Intracellular metabolite abundance of homocitrate. (D) Reaction catalyzed by homocitrate synthase. Homocitrate is then incorporated into the FeMo cofactor as a bidentate chelator of Mo.

Our proteomics analysis revealed a concerted increase in abundance of *nif* cluster proteins during N_2_-fixing conditions (Fig. 3B). Under continuous N_2_-fixing conditions, 27 out of 29 detected *nif* cluster proteins were significantly elevated (FC > 1.5, FDR < 0.05) (Fig. 3B). Levels of nitrogenase proteins NifH, NifD, and NifK were elevated by 23-fold, 41-fold, and 46-fold, respectively, during continuous N_2_ fixation (Fig. 4A). During the dynamic shift to N_2_-fixing conditions, 30 out of 31 *nif* cluster proteins increased significantly in abundance (Fig. 3B). Levels of NifH, NifD, and NifK were elevated by 2-fold above the NH_4_^+^-replete baseline after 1.5 h (just as growth stalled) and by over 8-fold after 6 h (Fig. 4A).

One of most affected proteins from the *nif* cluster was homocitrate synthase (NifV), which was 300-fold more abundant during continuous N_2_ fixation and increased by 16-fold during the shift to N_2_-fixing conditions (Fig. 4B). NifV transfers an acetyl group from acetyl coenzyme A (acetyl-CoA) to α-ketoglutarate to produce homocitrate, which chelates the Mo atom in the FeMo-co at the active site of nitrogenase (Fig. 4D) (40, 41). Metabolomics analysis showed that intracellular homocitrate levels increased by over 60-fold during N_2_ fixation (both continuous N_2_ fixation and transition to N_2_ fixation), which was the largest increase in metabolite abundance observed in this study (Fig. 2 and 4C).

During NH_4_^+^ upshift, fewer changes in *nif* cluster protein abundance occurred (Fig. 3B). Only 14 of the 31 *nif* cluster proteins decreased significantly relative to N_2_-fixing controls. The largest decreases were in NifZ, NifU, NifB, NifW, NifV, NifE, NifN, and the iron-sulfur cluster assembly accessory protein ZMO1832, all of which decreased by over 2-fold (Fig. 3B and 4B). Given that less than one cell doubling occurred during the NH_4_^+^ upshift time course experiment, decreases over 2-fold imply active protein degradation rather than dilution by cell division. Many of the proteins that decreased in abundance are involved in the biogenesis of FeS or FeMo cofactors, including NifV, whose depletion coincided with an 8-fold drop in intracellular levels of homocitrate (Fig. 4C). Protein levels of nitrogenase proteins NifH, NifD, and NifK all showed decreasing trends during NH_4_^+^ upshift, but only NifK met our criteria (FC > 1.5, FDR < 0.05) for differential expression and all three remained within 2-fold of the N_2_-fixing baseline (Fig. 4A). Together, these results suggest that biogenesis of FeS and FeMo cofactors is rapidly repressed via protein degradation during an acute increase in NH_4_^+^ availability, even while NifHDK protein abundance remains relatively constant. Delaying degradation of nitrogenase may provide a fitness advantage in environments where bioavailable nitrogen is transiently available. Modulation of iron-sulfur cluster biogenesis and intracellular homocitrate levels may contribute to the regulation of nitrogenase activity during changes in NH_4_^+^ availability.

### Decreased abundance of flagellar and chemotaxis proteins during N_2_ fixation.

In the Z. mobilis genome, the genes involved in flagellar motility (ZMO0602-ZMO0652) and several genes related to chemotaxis signal transduction (ZMO0078-ZMO0085) are each organized within their own gene cluster (30, 42). Several flagellar proteins (FlgL, FlgK, FlgI, FlgD, FlgC, FlgB, FliF, FliI, FliK, FliO, FliD, and FliK) and chemotaxis proteins (CheY, CheD, CheA, and CheX) from these two gene clusters were significantly depleted (FC > 1.5, FDR < 0.05) during the transition to N_2_-fixing conditions (Fig. S2). Decreases in abundance of motility proteins was seen as early as 1 h into the NH_4_^+^ downshift. FlgI, FliF, FliI, CheR, CheA, and McpJ were also less abundant during continuous N_2_-fixing conditions (FC > 1.5, FDR < 0.05). No significant trends were observed in proteins from either gene cluster in response to NH_4_^+^ upshift.

Bacterial flagellar motors are powered by the translocation of ions across the inner membrane (43, 44). Others have observed decreased expression of flagellar genes in Z. mobilis under stress conditions that disrupt the maintenance of an electrochemical gradient across the membrane, such as low pH, osmotic stress, and high ethanol concentrations (45–47). This is in contrast to other environmental inhibitors such as oxygen and lignocellulosic toxins, which have been shown to increase expression of motility-related genes in Z. mobilis (48–50). Previous transcriptomics analysis of Z. mobilis both during adaptation to high glucose concentrations and in the presence of a quorum sensing autoinducer each showed that expression of flagellar genes decreased concomitantly with increased expression of genes from the *nif* cluster (20, 46). Given that the Rnf complex utilizes energy stored in the electrochemical gradient to drive production of reduced ferredoxin (the electron donor to nitrogenase reductase), decreased expression of flagellar proteins (and, presumably, decreased energy consumption by the flagellar motor) may be important for maintaining a robust electrochemical gradient during N_2_-fixing conditions.

### Dynamics of NH_4_^+^ assimilation via the GS-GOGAT cycle.

**FIG 5:**
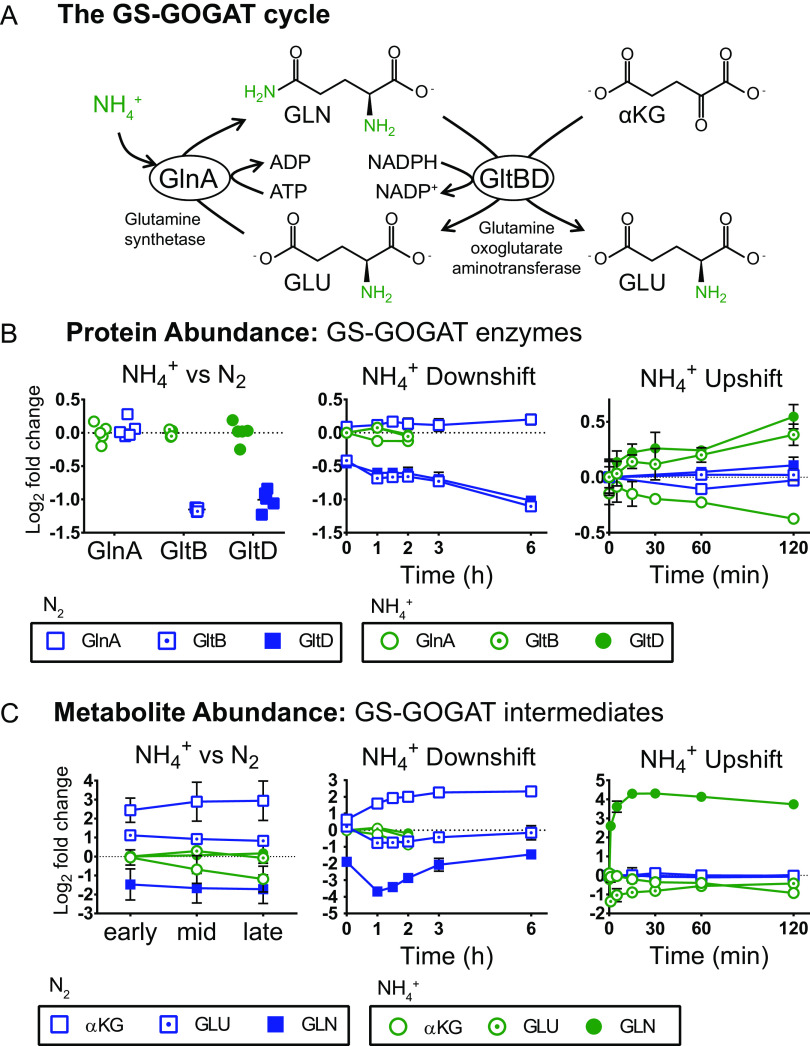
(A) Schematic of the GS-GOGAT cycle. (B and C) Relative abundance of proteins (B) and metabolites (C) in the GS-GOGAT cycle during three conditions of nitrogen availability. Blue squares indicate N_2_ fixation or transition to N_2_ fixation. Green circles indicate replete NH_4_^+^ or NH_4_^+^ upshift. Log_2_ fold change values are relative to the first time point in the control condition. For NH_4_^+^ versus N_2_ protein abundance, individual data points are shown (5 biological replicates per condition). For all other graphs, values are the average of at least 3 biological replicates for the treatment condition and at least 2 biological replicates for controls. Error bars show SD. (B) Protein abundance of glutamine synthetase (GlnA) (open symbols), the α (large) subunit of glutamine oxoglutarate aminotransferase (GltB) (open symbols with dots), and the β (small) subunit of GOGAT (GltD) (closed symbols). (C) Metabolite abundance of α-ketoglutarate (open symbols), glutamate (open symbols with dots), and glutamine (closed symbols).

There are two major pathways for NH_4_^+^ assimilation in bacteria (51). One of these pathways is comprised of glutamine synthetase (GS) and glutamine oxoglutarate aminotransferase (GOGAT), which together form the GS-GOGAT cycle (Fig. 5A). In this pathway, GS catalyzes the condensation of glutamate and ammonia to form glutamine, converting one molecule of ATP to ADP in the process (52). Next, GOGAT transfers the amino group from glutamine to α-ketoglutarate (αKG), forming two molecules of glutamate and converting one molecule of NAD(P)H to NAD(P)^+^ (53). The other bacterial route for ammonia assimilation is via glutamate dehydrogenase (GDH), which directly converts αKG to glutamate by reductive amination of αKG, also consuming one molecule of NAD(P)H (54). The two pathways differ in their energy (ATP) consumption and affinity for NH_4_^+^ (54–56). In Escherichia coli, it is generally understood that the GS-GOGAT cycle is employed during low NH_4_^+^ availability while GDH is active during high NH_4_^+^ availability or energy limitation (56, 57). In Z. mobilis, no *gdh* gene has been identified based on sequence homology (58). However, genes encoding GS (*glnA*) and the large and small subunits of GOGAT (*gltB* and *gtlD*) have been annotated (30) (Fig. 5A).

Proteomics analysis determined relative abundance of GS (GlnA) and the large and small subunits of GOGAT (GltB and GltD) during continuous N_2_ fixation, NH_4_^+^ downshift, and NH_4_^+^ upshift (Fig. 5B). Interestingly, GS levels did not change significantly for any of the three conditions of NH_4_^+^ availability (FC < 1.3, FDR > 0.2) (Fig. 5B). However, both subunits of GOGAT were 2-fold lower during continuous N_2_ fixation than during NH_4_^+^-replete conditions (Fig. 5B). During NH_4_^+^ downshift, GOGAT levels were already lower than in NH_4_^+^-replete controls at the initial time point (before changes in growth rate were observed), and they continued to fall for the duration of the NH_4_^+^ downshift time course, reaching 2-fold below controls at the 6-h time point (Fig. 5C). There was an increasing trend for both subunits of GOGAT during NH_4_^+^ upshift (FDR = 0.072 and 0.029, respectively), but levels remained within 1.5-fold of controls (Fig. 5B). Overall, GS enzyme levels remained constant and GOGAT enzymes levels decreased during N_2_ fixation. Regulation of GS via posttranslational adenylylation by GS adenylyltransferase/adenylyl-removing enzyme (AT/AR) is common in other proteobacteria (59). However, no AT/AR gene has been annotated in the Z. mobilis genome. On the other hand, differential phosphorylation of both GS and GOGAT have been observed during N_2_ fixation in Z. mobilis, likely contributing to the regulation of GS-GOGAT cycle activity (60).

Metabolomics analysis produced relative intracellular abundance of glutamine, glutamate, and αKG during the three conditions of NH_4_^+^ availability (Fig. 5C). Under continuous N_2_-fixing conditions, intracellular glutamine levels were 3-fold lower, glutamate levels were 2-fold higher, and αKG levels were 8-fold higher than under NH_4_^+^-replete conditions (Fig. 5C). During NH_4_^+^ downshift, glutamine levels were already 3-fold lower than those of NH_4_^+^-replete controls at the initial time point and dropped to 12-fold below controls after 1 h, when growth began to stall. From the 1-h time point to the 6-h time point, intracellular glutamine levels rose, tracking with the rise in nitrogenase protein levels (Fig. 5C and 4A). Glutamate levels remained within 2-fold of control levels for the entire downshift time course but matched the trends seen in glutamine, dropping in the first hour and then increasing from 1 to 6 h. Levels of αKG increased for the duration of the downshift time course, reaching 5-fold above baseline after 6 h (Fig. 5C).

During NH_4_^+^ upshift, intracellular glutamine levels immediately increased, reaching 6-fold above the N_2_-fixing control within 1 min of NH_4_^+^ addition. Glutamine levels continued to rise for 30 min following NH_4_^+^ addition, reaching 20-fold above baseline before gradually decreasing to 13-fold above baseline by the 2-h time point. Glutamate levels trended in the opposite direction, first dropping to 2.5-fold below N_2_-fixing controls within 1 min and then increasing for the remainder of the time course, ending within 1.5-fold of the controls. αKG levels did not change within the first 15 min of the NH_4_^+^ upshift time course but decreased by around 2-fold from 15 min to 2 h (Fig. 5C).

Previous studies in E. coli and Rhodospirillum rubrum have found that upon addition of NH_4_^+^ to nitrogen-limited cultures, intracellular glutamine levels increased rapidly but then quickly declined within 5 to 10 min (61, 62). The decline in glutamine levels following their accumulation was associated with fast inactivation of GS via adenylylation by AT/AR (61, 63, 64). In Z. mobilis, glutamine levels rapidly increased following NH_4_^+^ upshift but did not decline in the first 30 min and remained substantially elevated compared to steady-state levels for over 2 h, suggesting that GS activity is not rapidly inhibited in response to increased NH_4_^+^ availability. The fact that the Z. mobilis genome apparently does not encode AT/AR may help explain the persistence of elevated glutamine levels following NH_4_^+^ addition in Z. mobilis. More research should be conducted to investigate the regulatory mechanisms that control GS activity in Z. mobilis, as they appear to be distinct from the classical model that has been well studied in other proteobacteria (65, 66).

### Addition of ^15^NH_4_^+^ to N_2_-fixing cultures shows immediate assimilation of exogenous NH_4_^+^ and possible residual nitrogenase activity.

**FIG 6:**
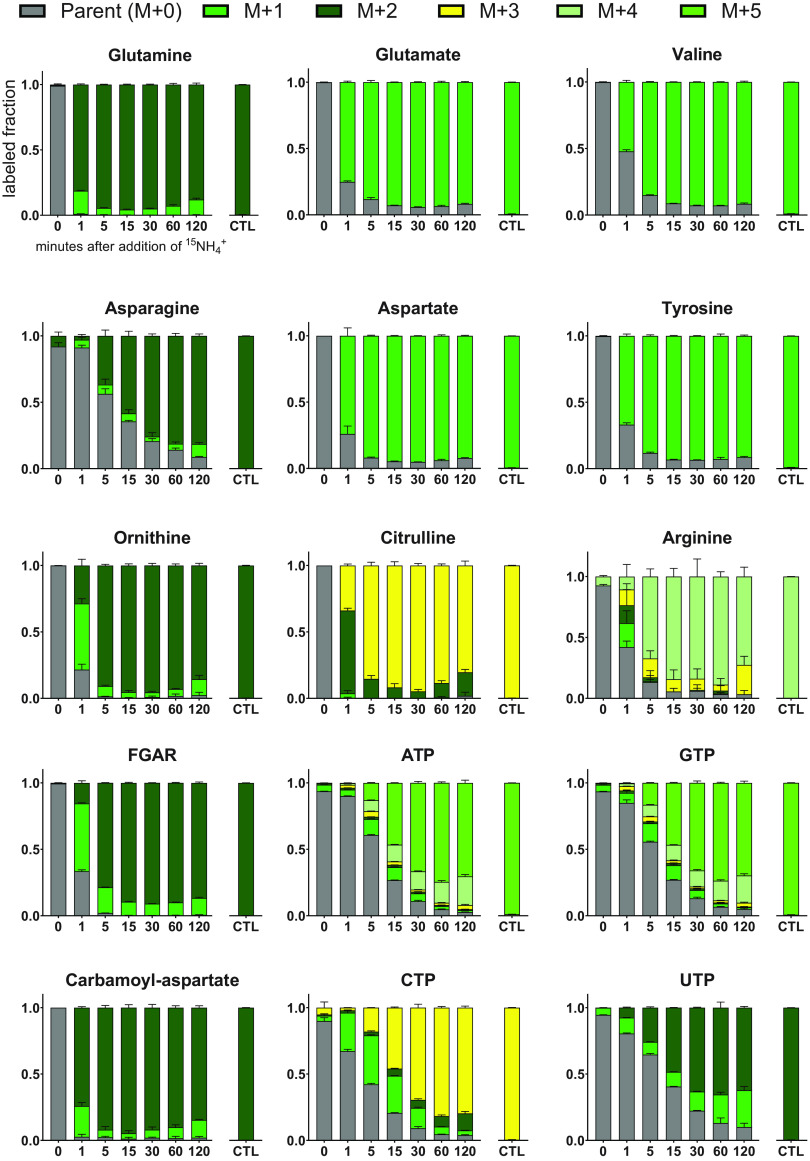
Dynamics of metabolite labeling patterns following addition of ^15^NH_4_^+^ to N_2_-fixing cultures (15 mM final concentration) compared to controls (CTL) that were grown with 15 mM ^15^NH_4_^+^ continuously. The *y* axis represents the relative proportion of each labeled form at each time point. Values are the average of 3 biological replicates. Error bars show SD. M + 1 indicates the presence of one ^15^N nitrogen atom in the molecule, M + 2 indicates the presence of two ^15^N nitrogen atoms, etc. M + 0 indicates that all nitrogen atoms are ^14^N. Masses were adjusted to account for the natural abundance of ^15^N (see Materials and Methods). Abbreviation: FGAR, phosphoribosyl-*N-*formylglycineamide.

The immediate increase in intracellular glutamine levels following NH_4_^+^ addition indicates that Z. mobilis is poised to rapidly incorporate exogenous NH_4_^+^ as soon as it becomes available, even in an N_2_-fixing state. This was expected given that NH_4_^+^ is a product of nitrogenase, and at high (mM) extracellular concentrations, NH_4_^+^ can passively diffuse across the membrane to support growth in other bacteria (67). To examine the dynamics of NH_4_^+^ assimilation and incorporation more closely, we performed a separate iteration of the NH_4_^+^ upshift experiment using ^15^NH_4_Cl (see Materials and Methods). This allowed us to trace the incorporation of exogenous NH_4_^+^ into metabolite pools, providing insight into the turnover rate of nitrogen-containing metabolites during NH_4_^+^ upshift (Fig. 6). Within 1 min after addition of ^15^NH_4_^+^, glutamine levels were 99% ^15^N labeled (i.e., containing at least one ^15^N atom). Similar trends were observed for glutamate, which was 90% ^15^N labeled after 5 min. Most amino acids followed these trends, with a few exceptions. Most notably, asparagine labeled much more slowly and was still over 50% unlabeled after 5 min. This suggests that either rates of asparagine biosynthesis are low compared to other amino acids, intracellular pools of asparagine are high compared to other amino acids, or there is some compartmentalized pool of asparagine (e.g., periplasmic or extracellular) that is subject to a lower turnover rate. The rate of ^15^N incorporation into nucleotide biosynthetic intermediates was similar to that of glutamine and glutamate, showing that exogenously supplied NH_4_^+^ was rapidly utilized for *de novo* synthesis of both purines and pyrimidines. Nucleotide triphosphates such as ATP labeled more slowly than their upstream intermediates, as would be expected, but after 2 h, less than 5% of the NTP pool remained unlabeled.

Interestingly, from 15 min to 2 h, we observed a small but significant (*P* < 0.01) loss of ^15^N label in glutamine. The fully labeled fraction (i.e., containing two ^15^N atoms) of glutamine went from 95% after 15 min to 88% after 2 h, implying increasing assimilation of unlabeled (^14^N) nitrogen. Loss of ^15^N label was also seen in other metabolites, including intermediates in amino acid and nucleotide biosynthesis. This loss of ^15^N label might be the result of protein degradation, which could liberate amino acids and NH_4_^+^ from proteins that had been translated during ^14^N_2_ fixation. However, given the observation that nitrogenase protein levels did not decrease by more than 2-fold during NH_4_^+^ upshift, loss of ^15^N label could also be caused by residual nitrogenase activity following increased availability of NH_4_^+^. In some diazotrophic alphaproteobacteria (e.g., *R. rubrum*), NH_4_^+^ upshift induces rapid but reversible inactivation of NifH via ADP-ribosylation by DraT/DraG (68–70). However, neither *draT* nor *draG* homologues have been identified in the Z. mobilis genome. Control samples extracted from cells grown with replete ^15^NH_4_^+^ for over 6 generations were >99% fully ^15^N labeled for all nitrogen-containing metabolites detected (Fig. 6). This indicates that long-term replete NH_4_^+^ availability does result in complete repression of nitrogenase activity.

### Effect of nitrogen availability on amino acid abundance.

**FIG 7:**
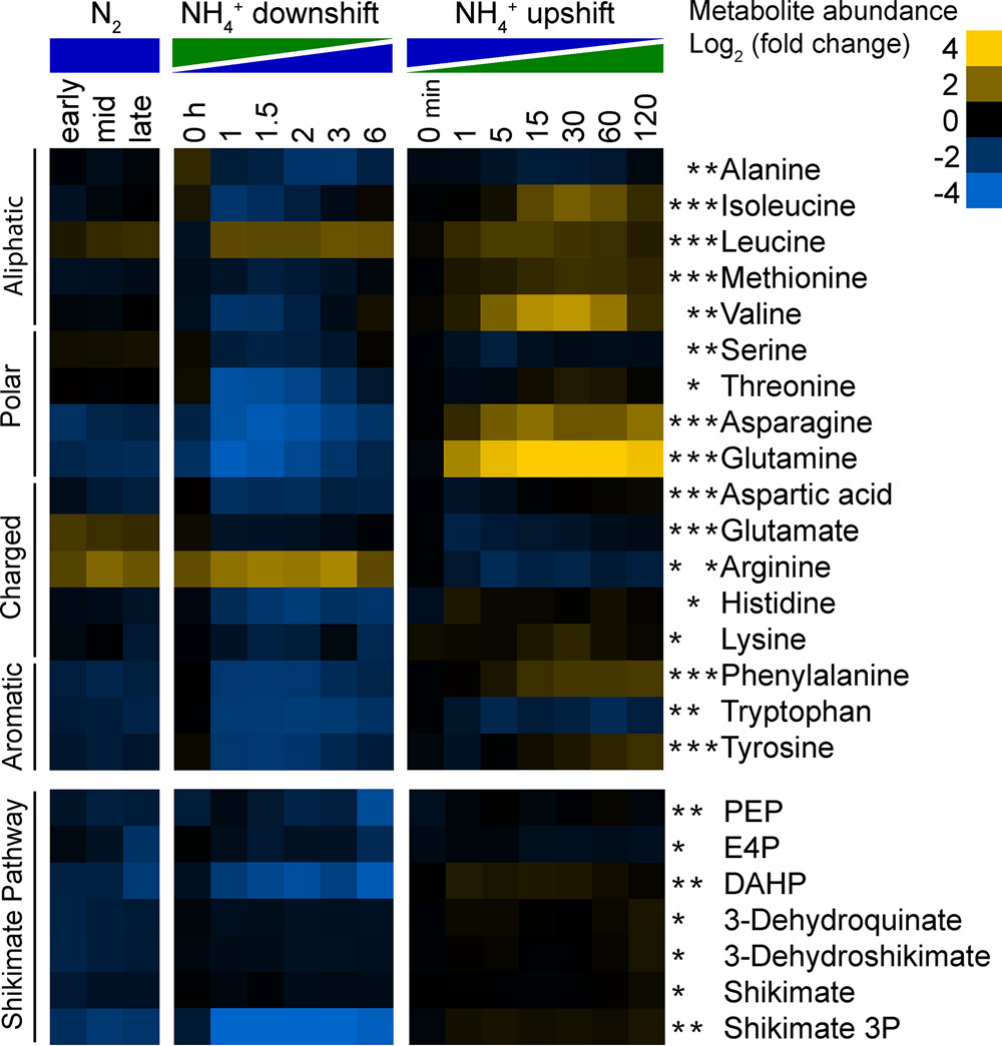
Relative intracellular metabolite abundance of amino acids and intermediates in aromatic amino acid biosynthesis. Log_2_ fold change values are relative to the first time point in the control condition (not shown). Values are the averages of at least 3 biological replicates. Asterisks indicate statistical significance (FC ≥ 1.5, FDR ≤ 0.05) for NH_4_^+^ versus N_2_, NH_4_^+^ downshift, and NH_4_^+^ upshift conditions, respectively, from left to right. For example, intracellular levels of alanine changed significantly during NH_4_^+^ downshift and NH_4_^+^ upshift but not during continuous NH_4_^+^ versus N_2_ conditions. Metabolites were manually arranged based on chemical properties of the side chain for amino acids and by metabolic pathway order for the shikimate pathway. Abbreviations: PEP, phosphoenolpyruvate; E4P, erythrose 4-phosphate; DAHP, 2-dehydro-3-deoxy-d-arabino-heptonate 7-phosphate.

We measured the relative intracellular abundance of 17 amino acids (Fig. 7). Levels of glutamine, asparagine, aspartic acid, isoleucine, methionine, lysine, and aromatic amino acids (phenylalanine, tryptophan, and tyrosine) were all significantly depleted (FC > 1.5, FDR < 0.05) during N_2_ fixation compared to NH_4_^+^-replete conditions. Glutamate, leucine, and arginine were the only amino acids whose levels were significantly elevated during continuous N_2_ fixation. During the dynamic shift to N_2_ fixation, all measured amino acids other than lysine, arginine, and leucine decreased significantly. Leucine and arginine levels both increased by over 3-fold, but lysine did not display any significant trends. During NH_4_^+^ upshift, the largest change in amino acid abundance was the 20-fold increase in glutamine levels, but asparagine, isoleucine, leucine, valine, methionine, tyrosine, and phenylalanine levels also increased significantly compared to the N_2_-fixing control. Abundance of glutamate, arginine, alanine, serine, and aspartic acid all decreased significantly during the NH_4_^+^ upshift.

### Depletion of aromatic amino acids and shikimate pathway intermediates during N_2_ fixation.

All three aromatic amino acids were significantly depleted under continuous N_2_-fixing conditions and during the dynamic shift to N_2_-fixing conditions. Intermediates of aromatic amino acid biosynthesis were also depleted under these conditions (Fig. 7). Of the seven intermediates of the shikimate pathway that we detected, all were found to be significantly depleted during continuous N_2_ fixation (FC > 1.5, FDR < 0.05). Additionally, levels of 2-dehydro-3-deoxy-d-arabino-heptonate 7-phosphate (DAHP), shikimate 3-phosphate, and (to a lesser extent) phosphoenolpyruvate (PEP) all decreased significantly during the shift to N_2_-fixing conditions. Shikimate 3-phosphate showed dramatic changes, dropping to 24-fold below NH_4_^+^-replete controls within the first hour. No changes were observed in levels of shikimate pathway intermediates during the NH_4_^+^ upshift.

### Arginine levels are elevated during N_2_ fixation despite depletion of intermediates in arginine biosynthesis.

**FIG 8:**
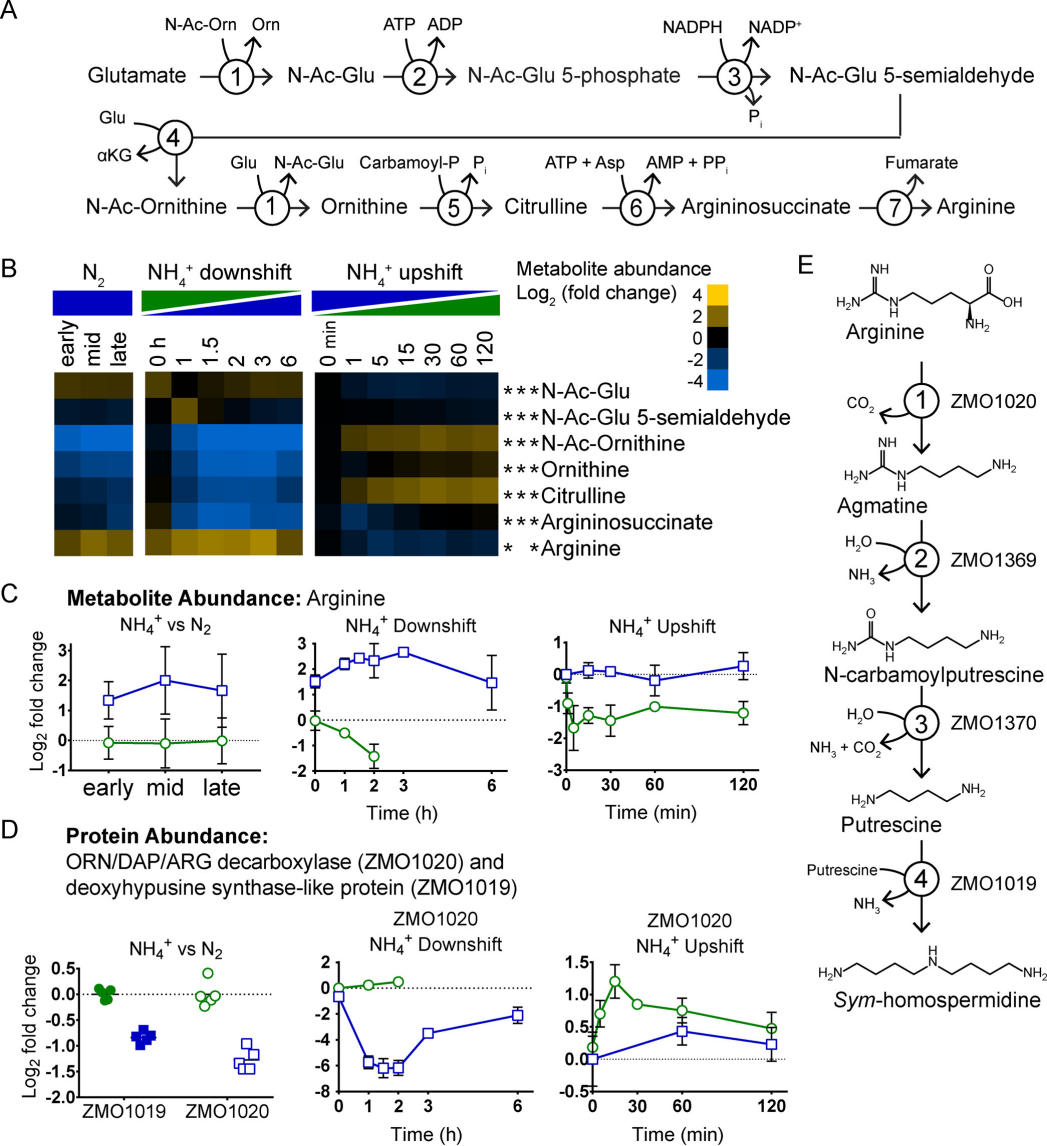
(A) Arginine biosynthetic pathway. 1, *N-*acetyl-ornithine:glutamate *N*-acetyltransferase (*argJ*); 2, *N*-acetylglutamate 5-phosphotransferase (*argB*); 3, *N*-acetylglutamate 5-semialdehyde dehydrogenase (*argC*); 4, *N*-acetylornithine 5-aminotransferase (*argG*); 5, carbamoylphosphate:ornithine carbamoyltransferase (*argF*); 6, argininosuccinate synthetase (*argG*); 7, argininosuccinase (*argH*). (B) Relative intracellular metabolite abundance of intermediates in arginine biosynthesis. Log_2_ fold change values are relative to the first time point in the control condition (not shown). Values are the averages of at least 3 biological replicates. Asterisks indicate statistical significance (FC ≥ 1.5, FDR ≤ 0.05) for NH_4_^+^ versus N_2_, NH_4_^+^ downshift, and NH_4_^+^ upshift conditions, respectively, from left to right. Metabolites were manually arranged by metabolic pathway order. (C) Relative intracellular metabolite abundance of arginine. (D) Relative protein abundance of ORN/DAP/ARG decarboxylase encoded by ZMO1020 and deoxyhypusine synthase-like protein encoded by ZMO1019. (C and D) Blue squares indicate N_2_ fixation (or transition to N_2_ fixation). Green circles indicate replete NH_4_^+^ (or NH_4_^+^ upshift). Log_2_ fold change values are relative to first time point in the control condition. For NH_4_^+^ versus N_2_ protein abundance, individual data points are shown (5 biological replicates per condition). For all other graphs, values are averages of at least 3 biological replicates for treatment condition and at least 2 biological replicates for control condition. Error bars show SD. (E) Hypothetical metabolic route from arginine to homospermidine via the enzymes encoded by ZMO1020, ZMO1019, ZMO1369, and ZMO1370. 1, arginine decarboxylase; 2, agmatine deiminase; 3, *N*-carbamoylputrescine amidase; 4, *sym*-homospermidine synthase.

Of the 17 measured amino acids, arginine was the only amino acid that increased during NH_4_^+^ downshift and decreased during NH_4_^+^ upshift (Fig. 7 and 8C). Arginine was also the only amino acid with levels over 4-fold higher during continuous N_2_ fixation than during NH_4_^+^-replete conditions. These results were surprising given that biosynthesis of arginine requires more nitrogen than any other amino acid and may therefore be expected to be depleted under conditions of nitrogen limitation. However, examination of intracellular levels of intermediates in arginine biosynthesis provides a potential explanation for this apparent contradiction. Intracellular levels of all intermediates of arginine biosynthesis downstream of *N-*acetyl glutamate 5-semialdehyde (i.e., *N-*acetyl ornithine, ornithine, citrulline, and argininosuccinate) were depleted during both continuous N_2_ fixation and the dynamic shift to N_2_ fixation (Fig. 8A and B). During NH_4_^+^ upshift, intracellular concentrations of *N-*acetyl ornithine, ornithine, and citrulline all increased by over 1.5-fold (Fig. 8B). During continuous N_2_ fixation, protein abundance of two enzymes in the arginine biosynthetic pathway (acetylglutamate kinase and argininosuccinate lyase) was significantly lower than in NH_4_^+^-replete controls (see Fig. S3 in the supplemental material). However, no significant changes were observed in protein abundance of arginine biosynthetic enzymes during NH_4_^+^ downshift or upshift. Taken together, these data suggest that arginine biosynthesis does decrease during N_2_ fixation. The fact that arginine still accumulates during N_2_ fixation implies a decreased rate of arginine consumption.

10.1128/mSystems.00987-21.3 FIG S3 Relative protein abundance of arginine biosynthetic enzymes (A) and nucleotide biosynthetic enzymes (B) during three conditions of nitrogen availability: from left to right, continuous NH_4_^+^-replete conditions versus continuous N_2_-fixing conditions, gradual NH_4_^+^ downshift, and acute NH_4_^+^ upshift, respectively. Rows are a single protein across all conditions, and columns are individual proteomics samples, taken at the indicated times and conditions. Log_2_ fold change values are relative to the first time point in the control condition (not shown). Values are the averages of at least 3 biological replicates. Yellow corresponds to increased protein abundance compared to the control, and blue indicates decreased protein abundance. Asterisks indicate statistical significance (FC ≥ 1.5, FDR ≤ 0.05) for NH_4_^+^ versus N_2_, NH_4_^+^ downshift, and NH_4_^+^ upshift conditions, respectively, from left to right. Proteins were manually arranged based on their position in their respective biosynthetic pathways. (C) Relative intracellular metabolite abundance of ribose 5-phosphate (R5P) and phosphoribosyl diphosphate (PRPP) under three conditions of nitrogen availability: from left to right, continuous NH_4_^+^-replete conditions versus continuous N_2_-fixing conditions, NH_4_^+^ downshift (shift to N_2_-fixing conditions), and NH_4_^+^ upshift (from N_2_-fixing conditions), respectively. Blue squares indicate N_2_ fixation or transition to N_2_ fixation. Green circles indicate replete NH_4_^+^ or NH_4_^+^ upshift. Log_2_ fold change values are relative to the first time point in the control condition. Values are the average of at least 3 biological replicates for treatment condition and at least 2 biological replicates for controls. Error bars show SD. Download FIG S3, EPS file, 1.6 MB. Copyright © 2021 Martien et al. 2021 Martien et al. https://creativecommons.org/licenses/by/4.0/ This content is distributed under the terms of the Creative Commons Attribution 4.0 International license.

### A potential pathway for *sym*-homospermidine biosynthesis from arginine.

Examination of proteomics data identified a candidate enzyme potentially responsible for differential arginine consumption during N_2_ fixation. Among the top 5 most differentially expressed proteins during the NH_4_^+^ downshift was a group IV decarboxylase, encoded by ZMO1020. Proteins of this family act as ornithine, diaminopimelate (DAP), or arginine decarboxylases (71). The ZMO1020 protein product decreased in abundance by 64-fold during the shift to N_2_-fixing conditions and remained severely depleted for 1 h (Fig. 8D). This severe depletion was somewhat transient, and after 6 h, protein levels were only 4-fold below the NH_4_^+^-replete baseline. During NH_4_^+^ upshift, ZMO1020 protein levels increased by over 2-fold within 15 min of NH_4_^+^ addition (Fig. 8B). Under continuous N_2_-fixing conditions, the ZMO1020 protein was 2-fold less abundant than under NH_4_^+^-replete conditions (Fig. 8D).

ZMO1020 is predicted to be in an operon with ZMO1019, which codes for a deoxyhypusine synthase-like protein (72). The ZMO1019 protein product was also depleted by around 2-fold during continuous N_2_ fixation, although it did not change significantly during dynamic shifts in NH_4_^+^ availability (Fig. 8D). Another operon in Z. mobilis is predicted to encode agmatine deiminase (ZMO1369) and *N-*carbamoyl putrescine amidase (ZMO1370). It has been demonstrated that the major polyamine produced in Z. mobilis is *sym*-homospermidine (73). The enzymes encoded by ZMO1020, ZMO1369, ZMO1370, and ZMO1019 form a feasible biosynthetic route from arginine to *sym*-homospermidine (Fig. 8E) (74–77). In this case, the deoxyhypusine synthase-like protein would function as a bacterial homospermidine synthase, combining two molecules of putrescine to generate *sym*-homospermidine. A similar pathway for *sym*-homospermidine production from arginine was identified in nitrogen-fixing cyanobacteria and was found to be required for robust diazotrophic growth (78). The negative correlation of arginine levels with ZMO1020 protein abundance, the organization of ZMO1020 and ZMO1019 genes within an operon, and the polyamine profile of Z. mobilis suggest that the protein encoded by ZMO1020 may function as an arginine decarboxylase, constituting the first step in *sym*-homospermidine biosynthesis in Z. mobilis. It is plausible that a shutdown of *sym*-homospermidine production during N_2_ fixation caused the observed accumulation of intracellular arginine. Accumulation of arginine may have then triggered the depletion of arginine biosynthetic intermediates via feedback inhibition, as has been well documented in other bacteria (79). More research is needed to confirm the activity of the proposed biosynthetic pathway for *sym*-homospermidine in Z. mobilis.

While the precise physiological role of polyamines in bacteria remains an open area of discovery, polyamine production has been implicated in the response to a variety of environmental stressors, including high temperatures, oxidative stress, and acidic conditions (80–82). In our previous study of oxygen exposure in Z. mobilis, intracellular levels of arginine, acetyl-ornithine, and ornithine followed trends during the shift to aerobic conditions very similar to those during the shift to N_2_-fixing conditions (Fig. S4) (48). Additionally, protein abundance of the potential arginine decarboxylase encoded by ZMO1020 dropped sharply in response to oxygen exposure, as it did in response to NH_4_^+^ depletion (Fig. S4). These observations suggest that the hypothesized production of *sym*-homospermidine from arginine may be regulated in response to a variety of environmental stressors.

10.1128/mSystems.00987-21.4 FIG S4 Relative intracellular metabolite abundance of acetyl-ornithine, ornithine, and arginine and relative protein abundance of group IV decarboxylase (ZMO1020) during NH_4_^+^ downshift (blue squares), compared to NH_4_^+^-replete controls (green circles), and during oxygen exposure (red circles), compared to anaerobic controls (gray squares) (oxygen exposure data from J. I. Martien, A. S. Hebert, D. M. Stevenson, M. R. Regner, et al., mSystems 4:e00284-18, 2019, https://doi.org/10.1128/mSystems.00284-18). Log_2_ fold change values are relative to the first time point in the control condition. Values are the average of at least 3 biological replicates for treatment condition and at least 2 biological replicates for controls. Error bars show SD. Download FIG S4, EPS file, 1.3 MB. Copyright © 2021 Martien et al. 2021 Martien et al. https://creativecommons.org/licenses/by/4.0/ This content is distributed under the terms of the Creative Commons Attribution 4.0 International license.

### Depletion of intermediates in nucleotide biosynthesis during N_2_ fixation.

**FIG 9:**
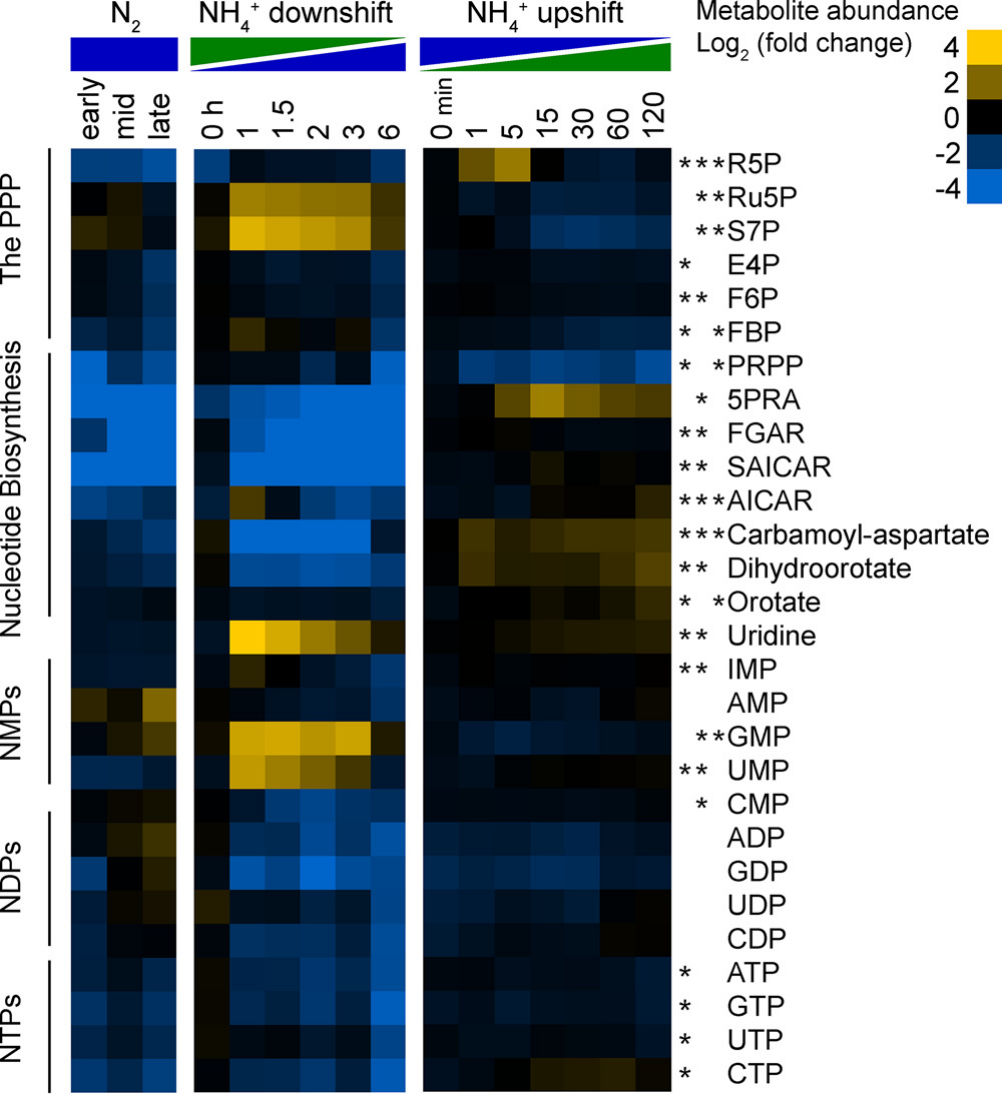
Relative intracellular metabolite abundance of nucleic acids and intermediates in nucleic acid biosynthesis. Log_2_ fold change values are relative to the first time point in the control condition (not shown). Values are the averages of at least 3 biological replicates. Asterisks indicate statistical significance (FC ≥ 1.5, FDR ≤ 0.05) for NH_4_^+^ versus N_2_, NH_4_^+^ downshift, and NH_4_^+^ upshift conditions, respectively, from left to right. For example, intracellular levels of S7P changed significantly for NH_4_^+^ downshift and NH_4_^+^ upshift only and E4P changed significantly only for NH_4_^+^ versus N_2_ conditions. Metabolites were manually arranged based on metabolic pathway and by number of phosphates for nucleotides. Abbreviations: R5P, ribose 5-phosphate; Ru5P, ribulose 5-phosphate; S7P, sedoheptulose 7-phosphate; E4P, erythrose 4-phosphate; F6P, fructose 6-phosphate; FBP, 1,6-fructose bisphosphate; PRPP, phosphoribosyl diphosphate; 5PRA, 5-phosphoribosylamine; FGAR, phosphoribosyl-*N*-formylglycineamide; SAICAR, phosphoribosylaminoimidazole-succinocarboxamide; AICAR, 5-aminoimidazole-4-carboxamide ribonucleotide.

Intermediates of *de novo* purine biosynthesis and *de novo* pyrimidine biosynthesis were depleted during both continuous N_2_ fixation and the shift to N_2_-fixing conditions (Fig. 9). Of the two pathways, purine biosynthesis was more severely impacted, with purine biosynthetic intermediates such as 5-phosphoribosylamine (5PRA), phosphoribosyl-*N-*formylglycineamide (FGAR), and phosphoribosylaminoimidazole-succinocarboxamide (SAICAR) reaching over 180-fold below NH_4_^+^-replete controls during the shift to N_2_ fixation. The largest change observed in intermediates of pyrimidine biosynthesis was in carbamoyl aspartate, which dropped to 30-fold below baseline during the shift to N_2_ fixation. During N_2_ fixation, there was a general depletion of nucleotide triphosphates and nucleotide diphosphates but an accumulation of nucleotide monophosphates and nucleosides. During the NH_4_^+^ upshift, both 5PRA and carbamoyl aspartate increased significantly but nucleotide levels remained unchanged. The 5-fold increase in intracellular 5PRA was accompanied by a 5-fold decrease in phosphoribosyl diphosphate (PRPP) levels within 15 min of addition of NH_4_^+^. This implies that amidophosphoribosyltransferase (PurF), which catalyzes the committed step in purine biosynthesis by incorporating an amino group from glutamine into PRPP to form 5PRA, is poised for rapid incorporation of nitrogen as soon as it becomes available (Fig. 9). This was consistent with rapid incorporation of ^15^N into FGAR following ^15^NH_4_^+^ addition (Fig. 6). We also observed dynamic changes in the PPP pathway following NH_4_^+^ upshift. In particular, ribose 5-phosphate (R5P) levels exhibited a sharp spike, reaching 5-fold above N_2_-fixing controls within 5 min of NH_4_Cl addition, only to drop back down to baseline by 15 min (Fig. S3). These trends highlight the tight regulation of PPP activity required to tune the supply of 5-carbon sugars for nucleotide biosynthesis during changes in nitrogen availability.

Despite the dramatic changes in intracellular metabolite levels of nucleotide biosynthetic intermediates, there were few significant changes in protein abundance of nucleotide biosynthetic enzymes during changes in NH_4_^+^ availability (Fig. S3). The only dynamic trend was a 1.7-fold decrease in PurF levels during the shift to N_2_ fixation. The metabolic activity of nucleotide biosynthesis therefore appears to be regulated via the availability of nitrogen-containing metabolic precursors (e.g., amino acids) rather than the abundance of metabolic enzymes.

### Depletion of MEP pathway intermediates and decreased abundance of DXS during N_2_ fixation.

**FIG 10:**
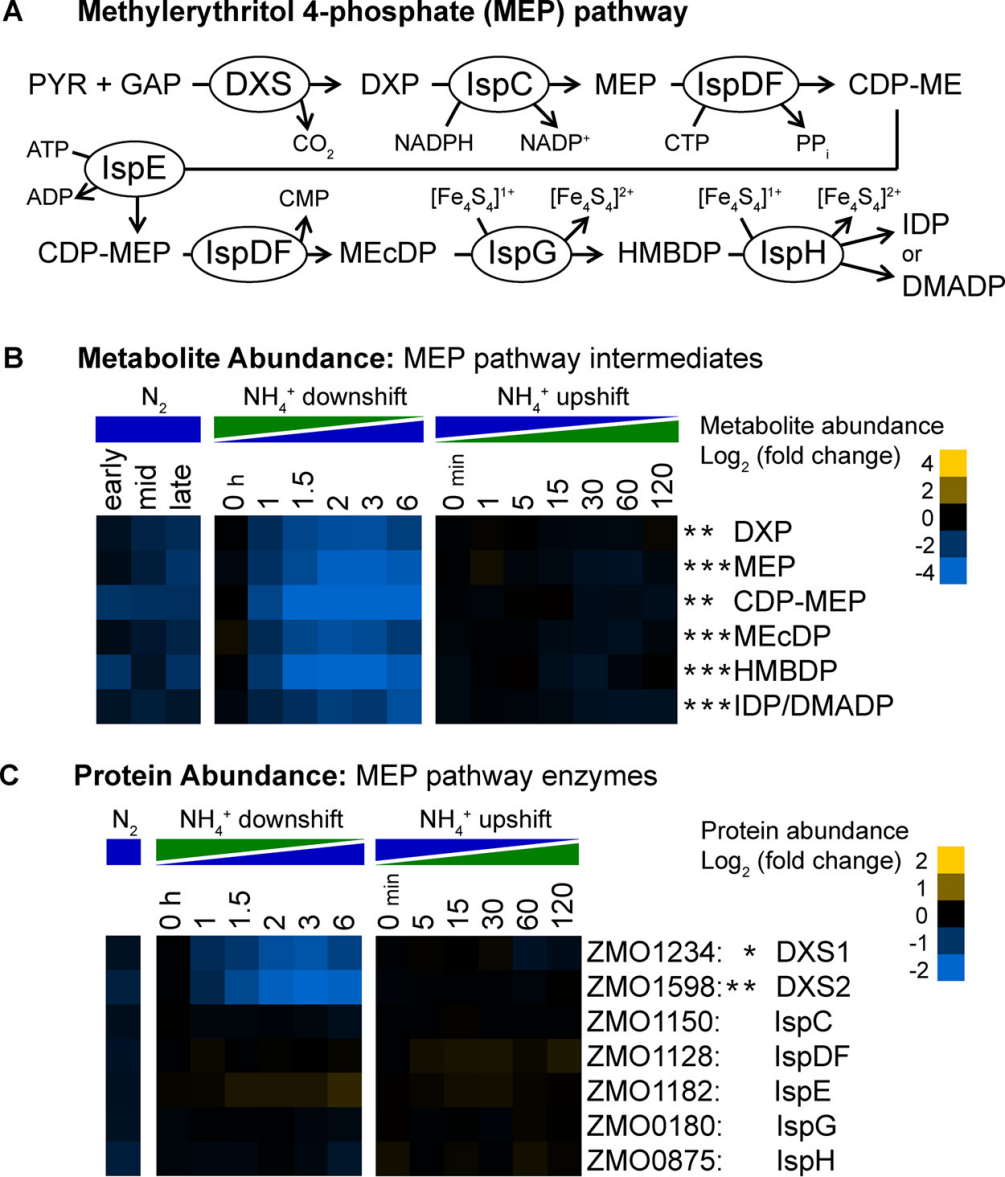
(A) Schematic of the MEP pathway. Abbreviations: PYR, pyruvate; GAP, glyceraldehyde 3-phosphate; DXP, 1-deoxy-d-xylulose 5-phosphate; MEP, 2-*C*-methylerythritol 4-phosphate; CDP-ME, 4-diphosphocytidyl-2-*C*-methyl-d-erythritol; CDP-MEP, 4-diphosphocytidyl-2-*C*-methyl-d-erythritol 2-phosphate; MEcDP, 2-*C*-methyl-d-erythritol 2,4-cyclodiphosphate; HMBDP, 4-hydroxy-3-methyl-butenyl 1-diphosphate; IDP, isopentenyl diphosphate; DMADP, dimethylallyl diphosphate; DXS, DXP synthase. (B) Relative intracellular metabolite abundance of intermediates in the MEP pathway. Isomers IDP and DMADP were detected as a single combined signal. The intermediate CDP-ME was below our level of detection. (C) Relative protein abundance of MEP pathway enzymes. DXS1 and DXS2 share 98% amino acid identity. (B and C) Log_2_ fold change values are relative to the first time point in the control condition (not shown). Values are the averages of at least 3 biological replicates. Yellow corresponds to increased metabolite abundance compared to the control, and blue indicates decreased metabolite abundance. Asterisks indicate statistical significance (FC ≥ 1.5, FDR ≤ 0.05) for NH_4_^+^ versus N_2_, NH_4_^+^ downshift, and NH_4_^+^ upshift conditions, respectively, from left to right. Metabolites and proteins were manually arranged by metabolic pathway order.

Z. mobilis exclusively utilizes the MEP pathway for the biosynthesis of isoprenoid precursors isopentenyl diphosphate (IDP) and dimethylallyl diphosphate (DMADP), which are required to produce biological compounds such as quinones and carotenoids (83). The MEP pathway starts with the condensation of pyruvate and glyceraldehyde 3-phosphate (GAP) to form 1-deoxy-d-xylulose 5-phosphate (DXP), catalyzed by DXP synthase (DXS). DXP is then converted to either IDP or DMADP via six enzymatic reactions carried out by IspC, IspD, IspE, IspF, IspG, and IspH (Fig. 10A). In Z. mobilis, there are two copies of the DXS enzyme (DXS1 and DXS2, encoded by ZMO1243 and ZMO1598, respectively) and IspD and IspF are fused and expressed as the single bifunctional enzyme IspDF.

Intracellular levels of all detected intermediates of the MEP pathway were lower during N_2_ fixation. Under continuous N_2_-fixing conditions, MEP pathway intermediates were between 2-fold and 4-fold lower than NH_4_^+^-replete controls (Fig. 10B). During the dynamic shift to N_2_ fixation, an even more pronounced depletion was observed. All detected MEP pathway intermediates decreased by between 8-fold and 32-fold during the 6-h NH_4_^+^ downshift time course (Fig. 10B). The intermediates 4-diphosphocytidyl-2-*C*-methyl-d-erythritol 2-phosphate (CDP-MEP) and 4-hydroxy-3-methyl-butenyl 1-diphosphate (HMBDP) displayed the largest decreases in abundance during both continuous N_2_ fixation and the shift to N_2_-fixing conditions. NH_4_^+^ upshift did not induce any increase in intracellular levels of MEP pathway intermediates, which all remained within 2-fold of the nitrogen-fixing control (Fig. 10B).

Of the seven enzymes of the MEP pathway, only the two DXS enzymes demonstrated significant changes in abundance in response to changes in NH_4_^+^ availability (Fig. 10C). All other MEP pathway enzymes remained within 1.5-fold of the control condition during all three nitrogen regimes. During continuous N_2_ fixation, DXS2 protein levels were significantly lower than NH_4_^+^-replete controls. During the NH_4_^+^ downshift experiment, both DXS1 and DXS2 decreased in abundance by over 3-fold within 3 h. However, following NH_4_^+^ upshift, neither DXS1 nor DXS2 changed in abundance during the 2-h time course.

Taken together, the trends in metabolite abundance of MEP pathway intermediates and protein abundance of MEP pathway enzymes suggest that MEP pathway activity is regulated during conditions of nitrogen limitation via abundance of the DXS enzyme. This is an interesting observation considering that activity of the MEP pathway does not directly consume nitrogen. The fitness benefit of regulating the MEP pathway in response to nitrogen availability may be related to the pathway’s consumption of ATP, CTP, or reduced cofactors. Additionally, decreased growth rates likely correspond to decreased demand for isoprenoid metabolites downstream of the MEP pathway. Others have reported experimental findings suggesting that the Clp protease complex may regulate MEP pathway activity in *Arabidopsis* plastids via degradation of DXS (84). More research is required to confirm targeted proteolytic degradation of DXS in Z. mobilis and to identify the mechanism of such degradation as well as the precise physiological cue, either nitrogen limitation itself or some secondary signal (for example depletion of NTP levels), that initiates the response.

### Depletion of ED pathway intermediates and increased abundance of ADHI during N_2_ fixation.

**FIG 11:**
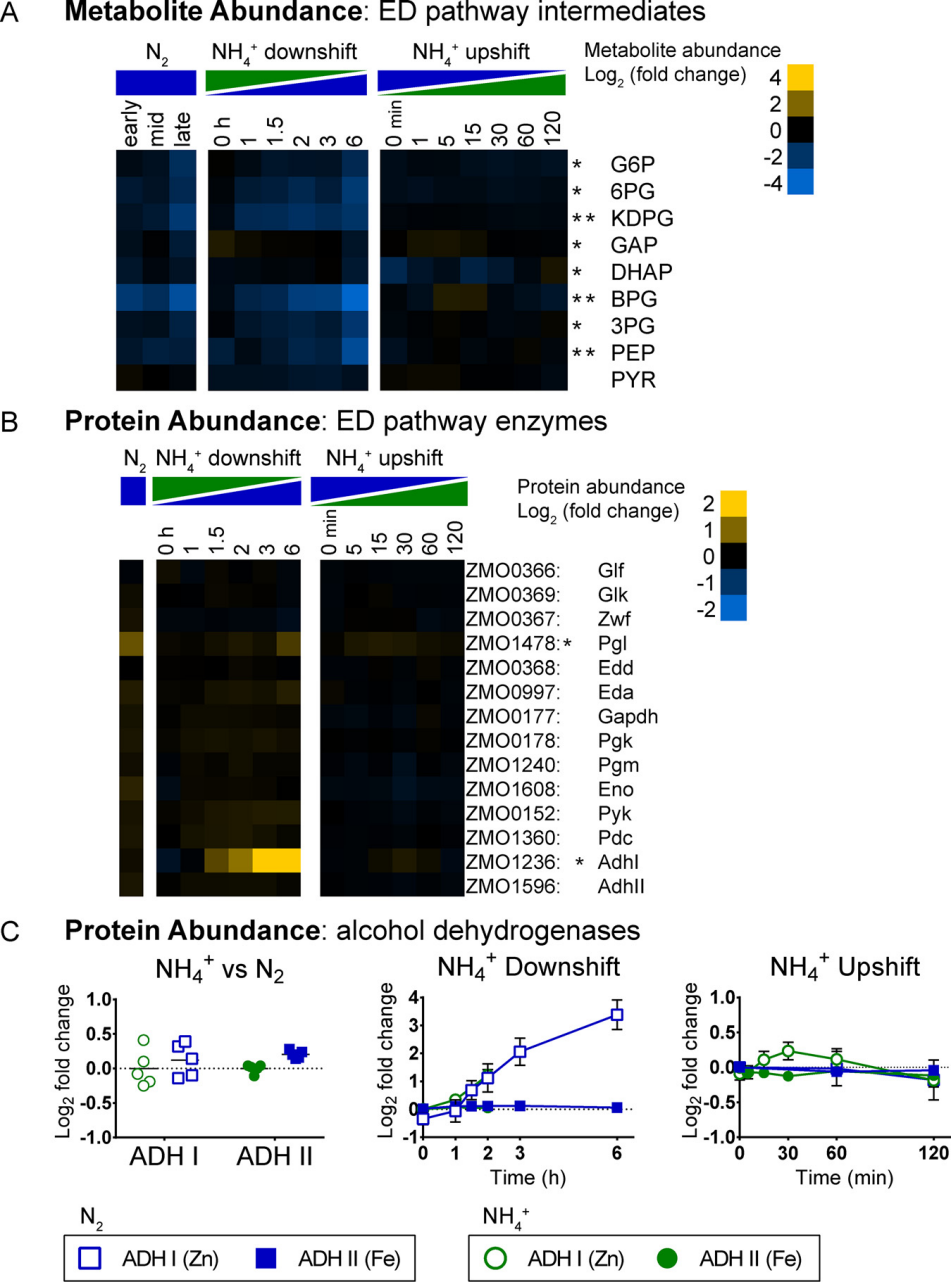
(A) Relative intracellular metabolite abundance of intermediates in the Entner-Doudoroff glycolytic pathway. Abbreviations: G6P, glucose 6-phosphate; 6PG, 6-phosphogluconate; KDPG, 2-dehydro-3-deoxy-d-gluconate 6-phosphate; GAP, glyceraldehyde 3-phosphate; DHAP, dihydroxyacetone phosphate; BPG, 1,3-bisphosphoglycerate; 3PG, 3-phosphoglycerate; PEP, phosphoenolpyruvate; PYR, pyruvate. (B) Protein abundance of ED pathway enzymes and alcohol dehydrogenases. (A and B) Log_2_ fold change values are relative to the first time point in the control condition (not shown). Values are the averages of at least 3 biological replicates. Asterisks indicate statistical significance (FC ≥ 1.5, FDR ≤ 0.05) for NH_4_^+^ versus N_2_, NH_4_^+^ downshift, and NH_4_^+^ upshift conditions, respectively, from left to right. Metabolites and proteins were manually arranged by metabolic pathway order. (C) Protein abundance of both Zn-dependent (ADHI, encoded by *adhA*) and Fe-dependent (ADHII, encoded by *adhB*) alcohol dehydrogenases. Blue squares indicate N_2_ fixation (or transition to N_2_ fixation). Green circles indicate replete NH_4_^+^ (or NH_4_^+^ upshift). Log_2_ fold changes are relative to the first time point in the control condition. For NH_4_^+^ versus N_2_ protein abundance, individual data points are shown (5 biological replicates per condition). For all other graphs, values are averages of at least 3 biological replicates for treatments and at least 2 biological replicates for the control condition. Error bars show SD. The only statistically significant change was in ADHI during NH_4_^+^ downshift.

Z. mobilis utilizes the ED pathway exclusively for glucose catabolism (85, 86). Intracellular concentrations of all quantified ED glycolytic intermediates were significantly depleted (FC > 1.5, FDR < 0.05) during continuous N_2_ fixation (Fig. 11A). Additionally, 2-keto-3-deoxy-6-phosphogluconate (KDPG), 1,3-bisphosphoglycerate (BPG), and phosphoenolpyruvate (PEP) levels decreased significantly during the shift to N_2_-fixing conditions (Fig. 11A). During the NH_4_^+^ upshift, no significant changes were observed for any intermediates of the ED pathway. Relative protein abundances of ED pathway enzymes were not significantly different during any of the three conditions of nitrogen availability, except for 6-phosphogluconolactonase (Pgl), which was 1.7-fold more abundant during continuous N_2_ fixation (Fig. 11B).

During N_2_ fixation, Z. mobilis exhibits increased specific rates of glucose consumption and ethanol production (17, 19, 20). However, the physiological factors that drive this response are not well understood. The Z. mobilis genome encodes two alcohol dehydrogenases, ADHI and ADHII. ADHI (encoded by *adhA*) is a zinc-dependent alcohol dehydrogenase, while ADHII (encoded by *adhB*) is iron dependent. Previous studies of purified ADHI and ADHII, and analysis of Δ*adhB* strains, indicate that both ADHI and ADHII contribute to ethanol production (87–91). We found that protein levels of ADHI increased by 10-fold during the shift to N_2_ fixation, placing it among the top 50 differentially abundant proteins observed in this study (Fig. 11C). No changes were observed in ADHI levels during continuous N_2_ fixation or during the NH_4_^+^ upshift. ADHII did not significantly change in abundance during changes in NH_4_^+^ availability (Fig. 11C). The difference in metal cofactors used by the two ADH isozymes is especially relevant considering that expression of nitrogenase holoenzyme significantly increases the cellular demand for iron. Increasing expression of zinc-dependent ADHI rather than iron-dependent ADHII may be important for maintaining iron homeostasis. Increased ADHI expression may help explain the previously reported increase in specific ethanol production during N_2_ fixation (17, 19, 20). However, the fact that no difference in ADHI abundance was detected during continuous N_2_-fixing conditions implies a possible difference in short-term and long-term mechanisms for increasing ethanol production.

### Increased thermodynamic favorability of the ED pathway during N_2_ fixation.

Thermodynamics constitutes a key determinant of flux and enzyme efficiency in metabolic networks. A pathway with a strong thermodynamic driving force (i.e., with an overall large negative change in Gibb’s free energy or Δ*G*) will achieve a higher net flux given a fixed amount of enzyme activity than one closer to equilibrium (86, 92, 93). Within a pathway, steps closer to equilibrium will be the least enzyme efficient. A reaction’s Δ*G* is related to its reversibility or reverse-to-forward flux ratio (*J*^−^/*J*^+^) by the equation Δ*G* = *RT* ln(*J*^−^/*J*^+^), where *R* is the gas constant and *T* is the temperature in Kelvin. *In vivo* reaction reversibility, and thus thermodynamics, can be examined using isotope tracers.

**FIG 12:**
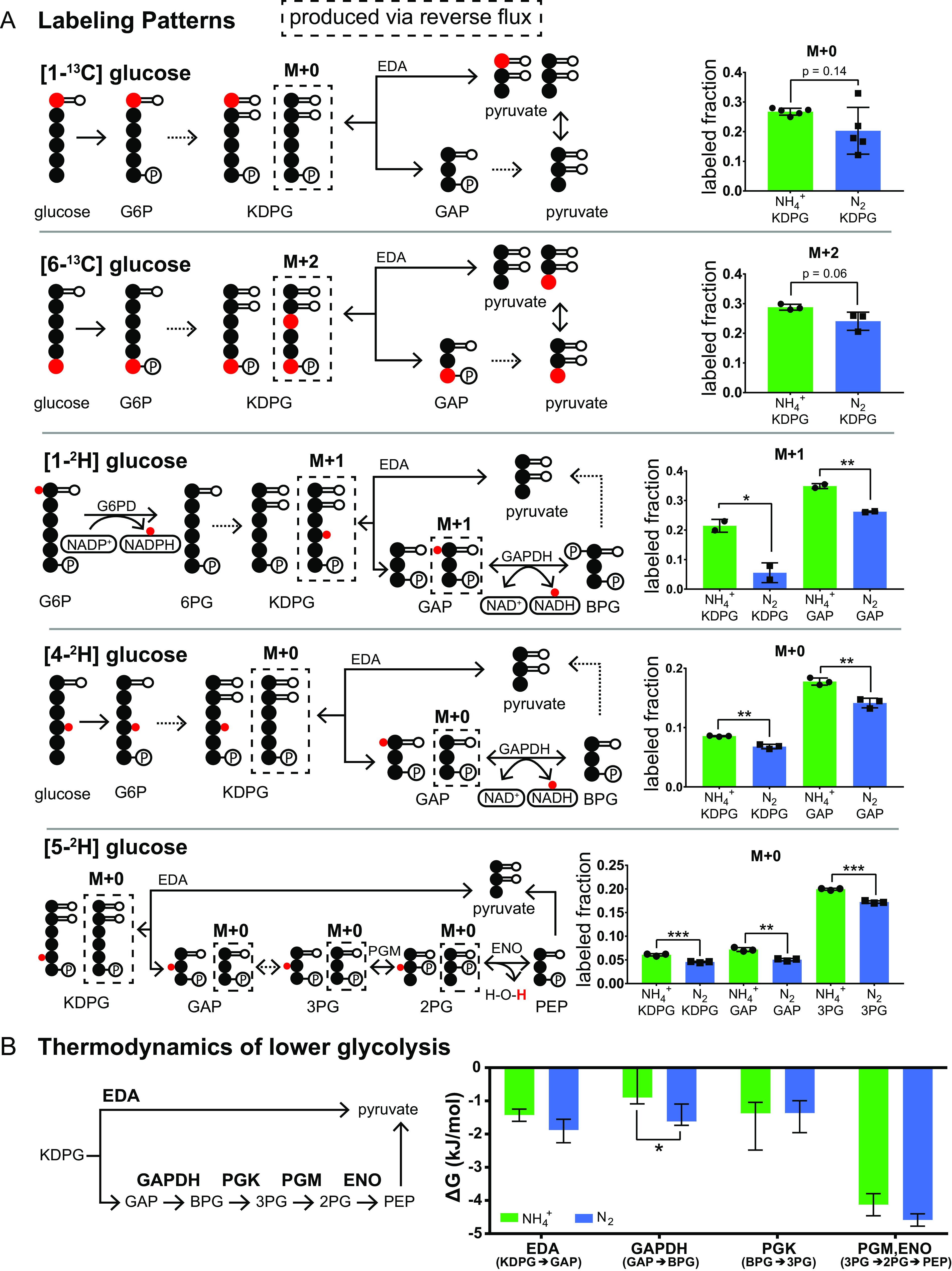
(A, left) Schematics of isotopic labeling patterns expected from forward and reverse flux through the ED pathway. Black circles represent unlabeled (^12^C) carbon atoms. Large red circles represent ^13^C-labeled carbon atoms. Small red circles represent ^2^H hydrogen (deuterium) atoms (^1^H hydrogens not shown). Dashed boxes surround the labeled form of an intermediate that can be generated only by reverse flux through the ED pathway. Dashed arrows at a step indicate multiple enzymatic steps and not-shown intermediates. (A, right) Experimental labeling patterns of ED pathway intermediates extracted from Z. mobilis grown on isotopically labeled glucose. The *y* axis is the fraction of the metabolite pool that has a mass/labeling pattern consistent with reverse flux (indicated by graph title). M + 1 indicates the presence of one ^13^C or one ^2^H atom in the molecule; M + 0 indicates that all atoms are ^12^C and ^1^H. Green bars show average fractions of metabolite generated by reverse flux from cells grown with replete NH_4_^+^, and blue bars show average fractions from cells grown under N_2_-fixing conditions. Individual samples are shown as black symbols. Error bars show SD. *P* values are from unpaired *t* tests comparing NH_4_^+^ with N_2_ for each metabolite. See Fig. S5 in the supplemental material for the complete labeling patterns of all ED intermediates. (B, left) Schematic of reactions in the lower ED glycolytic pathway. (B, right) Change in free energy (Δ*G*) for glycolytic reactions during N_2_ fixation (blue) or replete NH_4_^+^ availability (green). Δ*G* values are calculated based on forward-to-reverse flux ratios derived from metabolic flux analysis (MFA) using the equation Δ*G* = *RT* ln(*J*^−^/*J*^+^). Error bars represent 95% confidence intervals. Full results from the MFA model are reported in Table S4. Significance: *, *P* < 0.05; **, *P* < 0.01; ***, *P* < 0.001. Abbreviations: G6P, glucose 6-phosphate; 6PG, 6-phosphogluconate; KDPG, 2-dehydro-3-deoxy-d-gluconate 6-phosphate; GAP, glyceraldehyde 3-phosphate; BPG, 1,3-bisphosphoglycerate; 3PG, 3-phosphoglycerate; 2PG, 2-phosphoglycerate; PEP, phosphoenolpyruvate. Reactions: EDA, KDPG aldolase; GAPDH, GAP dehydrogenase; PGK, phosphoglycerate kinase; PGM, phosphoglycerate mutase; ENO, enolase (phosphopyruvate hydratase).

To examine the impact of NH_4_^+^ availability on ED pathway thermodynamics, we grew cells on glucose positionally labeled with either ^13^C or ^2^H under continuous N_2_-fixing conditions and NH_4_^+^-replete conditions. These experiments revealed that several reactions in the ED pathway were more reversible under NH_4_^+^-replete conditions, implying increased thermodynamic favorability of the ED pathway during N_2_ fixation (Fig. S5). In particular, labeling patterns of KDPG in cells fed either 100% [1-^13^C]glucose (98 to 99% isotopic purity) or 100% [6-^13^C]glucose (98 to 99% isotopic purity) indicated decreased reversibility of the KDPG aldolase reaction during N_2_ fixation (Fig. 12A). Labeling patterns of GAP from cells fed 100% [1-^2^H]glucose (98% isotopic purity) or 100% [4-^2^H]glucose (98% isotopic purity) were consistent with decreased reversibility of GAP dehydrogenase during N_2_ fixation (Fig. 12A). Labeling patterns of 3-phosphoglycerate (3PG) from cells fed 100% [5-^2^H]glucose (98% isotopic purity) were indicative of decreased reversibility of the two-step conversion of 3PG to PEP (Fig. 12A). Overall, the glycolytic reactions we observed were less reversible, and therefore more thermodynamically favorable, during N_2_ fixation.

10.1128/mSystems.00987-21.5 FIG S5 Experimental labeling patterns of ED pathway intermediates extracted from Z. mobilis grown on isotopically labeled glucose (as indicated in graph title) under continuous NH_4_^+^-replete conditions (left) or continuous N_2_-fixing conditions (right). The *y* axis is the fraction of the metabolite pool comprised of the different masses, indicated by different colors on the bar graph. M + 0 indicates that all atoms are ^12^C or ^1^H, M + 1 indicates the presence of one ^13^C or one ^2^H atom in the molecule, etc. Error bars show SD. Abbreviations: G6P, glucose 6-phosphate; 6PG, 6-phosphogluconate; KDPG, 2-dehydro-3-deoxy-d-gluconate 6-phosphate; GAP, glyceraldehyde 3-phosphate; DHAP, dihydroxyacetone phosphate; BPG, 1,3-bisphosphoglycerate; 3PG, 3-phosphoglycerate; PEP, phosphoenolpyruvate; PYR, pyruvate. Download FIG S5, EPS file, 1.2 MB. Copyright © 2021 Martien et al. 2021 Martien et al. https://creativecommons.org/licenses/by/4.0/ This content is distributed under the terms of the Creative Commons Attribution 4.0 International license.

10.1128/mSystems.00987-21.10 TABLE S4 Full results from metabolic flux analysis, including Δ*G* values, flux values, and atom mapping. Download Table S4, XLSX file, 0.02 MB. Copyright © 2021 Martien et al. 2021 Martien et al. https://creativecommons.org/licenses/by/4.0/ This content is distributed under the terms of the Creative Commons Attribution 4.0 International license.

To provide a quantitative estimate of the effect of N_2_ fixation on the thermodynamics of ED glycolysis, we fit glucose uptake rates, ethanol production rates, and labeling data from four ^13^C and ^2^H tracer experiments ([1-^13^C]glucose, [6-^13^C]glucose, [4-^2^H]glucose, and [5-^2^H]glucose) to a single flux map of ED glycolysis (Table S4). The equation Δ*G* = *RT* ln(*J*^−^/*J*^+^) was used to calculate reaction free energies for glycolytic reactions using forward-to-reverse flux ratios derived from metabolic flux analysis (MFA) (85, 94). For highly thermodynamically favorable reactions in upper ED glycolysis, i.e., glucose-6-phosphate 1-dehydrogenase (ZWF), 6-phosphogluconolactonase (PGL), and 6-phosphogluconate dehydratase (EDD), and the pyruvate kinase (PYK) reaction, forward-to-reverse flux ratios were not well resolved by MFA. Therefore, intracellular metabolite concentrations were used to provide tighter bounds on reaction free energies for these reactions, following the equation Δ*G* = Δ*G*°′ *RT* ln(*Q*), (where *Q* is the ratio of products to reactants) as previously described (85) (Table S4).

For highly favorable reactions (e.g., upper ED glycolysis), a small increase in Δ*G* has a minimal impact on net forward flux, whereas for reactions that are closer to equilibrium (e.g., lower ED glycolysis), a modest increase in Δ*G* can result in a substantial increase in net flux (92, 95). The MFA-derived free energies of lower ED pathway reactions are displayed in Fig. 12B and support the qualitative interpretation of the labeling data: glycolytic reactions tend to be more thermodynamically favorable during N_2_ fixation than when NH_4_^+^ is supplied in the medium. The glyceraldehyde-3-phosphate dehydrogenase (GAPDH:GAP + NAD^+^ + P_i_ → BPG + NADH + H^+^) reaction in particular was significantly more thermodynamically favorable during N_2_ fixation (*P* < 0.05). The optimal solution Δ*G* value of the GAPDH reaction was −1.617 kJ/mol during N_2_ fixation and −0.897 kJ/mol when NH_4_^+^ was provided. The increased thermodynamic favorability of GAPDH may be due to increased consumption of reducing power by nitrogenase, thereby depleting one of the products (i.e., NADH) of the GAPDH reaction and driving the reaction forward. Other ED pathway reactions did not display significantly different free energies (*P* > 0.05) but did trend toward greater thermodynamic favorability under N_2_-fixing conditions than under NH_4_^+^-replete conditions (Fig. 12B; Table S4).

Increased thermodynamic favorability of glycolysis during N_2_ fixation is a striking observation considering that the ED pathway in Z. mobilis is already highly thermodynamically favorable under NH_4_^+^-replete conditions (85). High thermodynamic favorability of glycolysis has been proposed to contribute to high rates of glucose consumption (85, 86). A previous study found that in E. coli, thermodynamic favorability of the Embden-Meyerhof-Parnas (EMP) glycolytic pathway increased during an upshift in nitrogen or phosphorus availability, coinciding with an increased rate of glucose uptake (95). In Z. mobilis, increased thermodynamic favorability of the ED pathway may thus contribute to increased rates of glucose consumption and ethanol production.

### Conclusions. (i) Regulation of nitrogenase activity in Z. mobilis.

The results of our metabolomics and proteomics analysis indicate that acute NH_4_^+^ upshift induces decreased production of nitrogenase cofactors (e.g., FeS clusters, homocitrate) but not immediate, pronounced degradation of nitrogenase itself (Fig. 3 and 4). The decreased abundance of proteins involved in cofactor biogenesis (e.g., NifZ, NifB, NifU) and the decline in intracellular levels of homocitrate during NH_4_^+^ upshift may play a role in downregulating nitrogenase activity. Proteolytic degradation of NifB, NifE, and NifN has been observed in Azotobacter vinelandii under N_2_-fixing conditions and was found to be important for iron conservation (96). In Z. mobilis, rapid degradation of proteins involved in nitrogenase cofactor biogenesis in response to NH_4_^+^ upshift may help to optimize iron utilization as soon as nitrogenase activity is no longer required.

Decreased biogenesis of nitrogenase cofactors may inhibit maturation or repair of new or damaged nitrogenase complexes but is not expected to inactivate holoenzyme. Indeed, tracer analysis using ^15^NH_4_^+^ indicated that residual nitrogenase activity may persist for at least 2 h following NH_4_^+^ upshift (Fig. 6). However, based on labeling patterns, only a small fraction of assimilated nitrogen is derived from non-^15^NH_4_^+^ sources following NH_4_^+^ upshift, suggesting that if residual nitrogenase activity persists, it is inhibited compared to its fully active state (Fig. 6). It is possible that posttranslational modification plays a role in reversibly inactivating nitrogenase during NH_4_^+^ upshift, as has been demonstrated in other N_2_-fixing bacteria (68–70). Differential phosphorylation of NifH and NifD during N_2_ fixation has been observed in Z. mobilis (60). However, *draT* and *draG*, the genes responsible for reversible inactivation of NifH via ADP-ribosylation in other organisms, have not been annotated in the Z. mobilis genome (30).

We previously reported a multi-omics analysis of oxygen exposure in Z. mobilis (48). Interestingly, unlike during the NH_4_^+^ upshift, pronounced degradation of nitrogenase proteins did occur when cells were rapidly transferred from anaerobic to aerobic conditions during exponential growth. NifH, NifD, and NifK levels dropped to between 5-fold and 45-fold below anaerobic baseline within 2 h after oxygen exposure (48). The oxygen exposure time course was performed under NH_4_^+^-replete conditions, so the initial abundance of nitrogenase was lower than it was at the beginning of the NH_4_^+^ upshift time course. However, the relative decrease in nitrogenase protein was much more pronounced during oxygen exposure than it was during NH_4_^+^ upshift. Z. mobilis cultures immediately stop growing following a shift to aerobic conditions when NH_4_^+^ is not supplied in the medium, suggesting that Z. mobilis lacks the ability to protect nitrogenase from oxidative damage (Fig. S6). A regulatory mechanism that stimulates high rates of nitrogenase protein degradation during oxygen exposure but delays complete degradation during NH_4_^+^ upshift would therefore prevent detrimental accumulation of damaged nitrogenase while potentially allowing for rapid adaptation to fluctuating NH_4_^+^ availability.

10.1128/mSystems.00987-21.6 FIG S6 Representative growth of Z. mobilis cultures measured by optical density at 600 nm during oxygen exposure (open symbols) or under continuous anaerobic conditions (closed symbols) grown with 0 mM NH_4_^+^ (N_2_ fixing; blue squares) or 15 mM NH_4_^+^ (NH_4_^+^ replete; green circles). Oxygen exposure was conducted at the times indicated by black arrows. Z. mobilis was grown at 30°C in an anaerobic chamber containing 5% CO_2_, 1 to 3% H_2_, and the remaining percentage N_2_. Flasks exposed to O_2_ were transferred out of the anaerobic chamber and shaken under atmospheric conditions for the remainder of growth. Download FIG S6, EPS file, 0.7 MB. Copyright © 2021 Martien et al. 2021 Martien et al. https://creativecommons.org/licenses/by/4.0/ This content is distributed under the terms of the Creative Commons Attribution 4.0 International license.

### (ii) Increased glycolytic activity may help meet increased energy demands associated with N_2_ fixation.

Regulation of carbon metabolism during changes in nitrogen availability is required to maintain the optimal ratio of carbon to nitrogen for biomass production. For most model microorganisms (e.g., Escherichia coli, Bacillus subtilis, Saccharomyces cerevisiae), decreased nitrogen availability results in a decreased rate of carbon uptake (97–99). However, for Z. mobilis, the opposite is true; nitrogen limitation results in increased specific glucose consumption despite decreased growth yield (17, 19, 100). In this study, we provide insight into the potential mechanisms driving this metabolic response, including decreased activity of biosynthetic pathways, increased protein abundance of ADHI, and increased thermodynamic favorability of the ED pathway during N_2_ fixation. The metabolic strategy for balancing carbon and nitrogen levels in Z. mobilis appears to leverage redirecting glucose toward ethanol production and away from biosynthetic reactions rather than decreasing glucose uptake. For Z. mobilis, glucose catabolism via the ED pathway is the only means of producing ATP, which is required to power N_2_ fixation by nitrogenase. Increased ED pathway activity may therefore be required to meet the high energy demands of N_2_ fixation. Additionally, the resulting increase in biomass-specific glucose consumption and ethanol production may serve a competitive advantage in the glucose-rich ecological niches to which Z. mobilis is adapted (101).

A recent publication examined the effect of type 2 quorum sensing autoinducer (AI-2) on Z. mobilis physiology. Because Z. mobilis does not synthesize AI-2, its presence can be interpreted as an indication of the proliferation of competing microorganisms. The study found that AI-2 induced increased nitrogenase activity together with increased biomass-specific glucose consumption and ethanol production by Z. mobilis (20). Interestingly, this study also found that ED pathway genes were not consistently differentially transcribed in the presence of AI-2, but several carbohydrate transporters were. We did not observe any consistent increases in protein abundance of carbohydrate transporters during N_2_ fixation in this study (Table S2). The glucose-facilitated diffusion protein (Glf) encoded directly upstream of the operon containing glucose-6-phosphate 1-dehydrogenase (*zwf*), phosphogluconate dehydratase (*edd*), and glucokinase (*glk*) genes was not differentially abundant at the protein level during any conditions of NH_4_^+^ availability (Fig. 11B) (102). Increased thermodynamic favorability of the ED pathway helps to explain the increase in biomass-specific glucose consumption rates during N_2_ fixation despite no increases in the abundance of glucose transporters or glycolytic enzymes. Nitrogenase activity may directly contribute to the increased thermodynamic favorability of the ED pathway by consuming both energy (ATP) and reducing equivalents [NAD(P)H], two of the products of glycolysis. We found that intracellular levels of all NTPs were significantly lower under continuous N_2_-fixing conditions (Fig. 9). It is therefore possible that N_2_-fixing conditions result in increased specific rates of glucose consumption and ethanol production by reducing intracellular ATP and NAD(P)H concentrations, thereby driving increased flux through the ED pathway via thermodynamic control.

### (iii) Implications for biofuel production.

The results presented in this study provide valuable insight into the native regulation of metabolic pathways required for biofuel production in Z. mobilis. In particular, the MEP pathway is a metabolic engineering target for microbial production of isoprenoid bioproducts, including transport fuels, polymers, pharmaceuticals, fragrances, and flavor additives (103). Decreased concentrations of MEP pathway intermediates coincided with decreased abundance of DXS protein. The rate of DXS protein depletion was higher than the growth rate, implying active protein degradation rather than dilution by cell division. This suggests that DXS degradation natively inhibits MEP pathway activity in Z. mobilis, as has been proposed for *Arabidopsis* plastids (84). This finding has significant ramifications for metabolic engineering efforts to develop Z. mobilis as a platform organism for isoprenoid production. Metabolic engineering strategies often rely on transcription-based control of gene expression with the assumption that increased transcription will result in increased protein abundance. It will therefore be important to further investigate the mechanism of DXS protein degradation in order to avoid degradation-driven repression of MEP pathway activity in engineered strains.

The observation that the abundance of motility proteins decreased during N_2_ fixation provides an interesting insight into native mechanisms for energy conservation in Z. mobilis. The impact of flagellar motility on the electrochemical gradient across the inner membrane is an important factor to consider for metabolic engineering. It is possible that deletion of motility genes may help conserve cellular energy, which could be directed to energy-intensive biofuel-producing pathways such as the MEP pathway.

Aside from isoprenoids, many bioproducts such as acetone, isobutanol, and butanediol are derived from the products of glycolysis and compete with ethanol production for substrates. Understanding the native regulation of glycolysis and ethanol production is therefore critical in optimizing the production of these biofuels in a microbial system. Previous studies showed that specific ethanol production and specific glucose uptake both increased during N_2_ fixation in Z. mobilis (17, 19, 20). In this study, we showed that the thermodynamic favorability of the ED glycolytic pathway increased during N_2_ fixation and that the protein abundance of zinc-dependent alcohol dehydrogenase ADHI increased during NH_4_^+^ downshift. These findings help elucidate the underlying physiological mechanisms that drive changes in carbon utilization in Z. mobilis and will help inform metabolic engineering for increased conversion of sugars to bioproducts.

Finally, optimized performance of microbial biofuel producers requires not only maximal product generation but also robust and consistent growth despite the presence of diverse chemical inhibitors. Polyamines have long been appreciated to participate in the bacterial response to environmental stress, but the metabolic pathway for polyamine production in Z. mobilis is still unknown. Accumulation of arginine despite depletion of arginine biosynthetic intermediates suggests that polyamine production from arginine may be repressed during the transition to N_2_-fixing conditions. Understanding the mechanism behind this metabolic response, and the physiological role of *sym*-homospermidine biosynthesis in stress tolerance, will therefore likely be informative in developing a robust biofuel-producing strain of Z. mobilis.

## MATERIALS AND METHODS

### Medium preparation.

To prepare the liquid minimal medium used in this study, a 10× glucose solution (200 g/liter), 10× base solution [10 g/liter KH_2_PO_4_, 10 g/liter K_2_HPO_4_, 5 g/liter NaCl, and either 10 g/liter (NH_4_)_2_SO_4_ or 2 g/liter MgSO_4_], and 1,000× solutions of MgSO_4_·7 H_2_O (200 g/liter), Na_2_MoO_4_·2 H_2_O (25 g/liter), and CaCl_2_ (10 g/liter) were prepared and autoclaved separately. One thousand-fold solutions of FeSO_4_ (2.5 g/liter) and calcium pantothenate (1 g/liter) were also prepared separately and filter sterilized using a 0.2-μm-pore-size filter. Autoclaved deionized water was then aseptically combined with the separately sterilized solutions to produce 1× concentrations of the added components. Finally, pH was measured to ensure that the medium was within the pH 6 to 6.5 range.

### Culture conditions.

Zymomonas mobilis subsp. *mobilis* strain ZM4 (ATCC 31821) was struck onto rich medium-glucose plates (20 g/liter glucose, 2 g/liter KH_2_PO_4_, 10 g/liter yeast extract, 18 g/liter agar) from a frozen 25% glycerol stock and incubated at 30°C in an anaerobic chamber for 3 to 6 days. For each biological replicate, a single colony was used to inoculate a flask of liquid minimal medium with replete ammonia [20 g/liter glucose, 1 g/liter KH_2_PO_4_, 1 g/liter K_2_HPO_4_, 0.5 g/liter NaCl, 1 g/liter (NH_4_)_2_SO_4_, 0.2 g/liter MgSO_4_·7 H_2_O, 25 mg/liter Na_2_MoO_4_·2 H_2_O, 2.5 mg/liter FeSO_4_·7 H_2_O, 0.01 g/liter CaCl_2_·2 H_2_O, 1 mg/liter calcium pantothenate]. After 14 to 16 h of growth, a small volume (10 μl to 1 ml) of this culture was used to inoculate subsequent cultures which contained either 15 mM NH_4_^+^ or no NH_4_^+^. In the case of no-NH_4_^+^ minimal medium, the 1 g/liter (NH_4_)_2_SO_4_ was replaced with 0.2 g/liter MgSO_4_ to achieve similar molarity. All cultures were grown in foil-covered Erlenmeyer flasks with an approximately 1:5 ratio of liquid volume-to-flask capacity. The total liquid volume ranged from 25 ml (in 125-ml flasks) to 100 ml (in 500-ml flasks). Cultures were stirred with a magnetic stir bar set to 120 rpm. All medium was kept anaerobic for at least 16 h prior to inoculation. The atmosphere in the anaerobic chamber was composed of 2 to 4% H_2_, 5% CO_2_, and the remaining percentage N_2_. Oxygen levels were kept below 100 ppm.

### Comparison of N_2_ and NH_4_^+^.

To compare continuous N_2_ fixation to NH_4_^+^-replete conditions, each of three separate NH_4_^+^-replete starting cultures were used to inoculate minimal medium with 15 mM NH_4_^+^ (replete) and minimal medium with no NH_4_^+^ (N_2_-fixing conditions), resulting in three biological replicates per condition. These cultures were grown anaerobically for up to 16 h. Before reaching stationary phase, cultures were used to inoculate fresh medium, maintaining the same NH_4_^+^ availability. Again, before reaching stationary phase, the second set of cultures was used to inoculate experimental flasks with a starting optical density at 600 nm (OD_600_) of approximately 0.05. This passaging was performed both to ensure continuous NH_4_^+^ availability and to dilute any nutrients provided by the rich medium plate. The result was that experimental cultures for the N_2_-fixing condition were inoculated with cells that had been growing under N_2_-fixing conditions for at least 6 doublings. Following inoculation of experimental cultures, extractions were performed for intracellular metabolite analysis during early, mid-, and late exponential phase (OD_600_ of 0.3, 0.5, and 0.7, respectively) for both NH_4_^+^-replete and N_2_-fixing cultures. For proteomics, a separate experiment was conducted with the same culture inoculation protocol except that there were five replicates instead of three. Extractions for proteomic analysis were performed at mid-exponential phase (OD_600_ of 0.5) only.

### NH_4_^+^ downshift.

To examine the transition to N_2_-fixing conditions, two rounds of passaging were performed as described for the N_2_ versus NH_4_^+^ experiment, except that all passaging was done in medium containing 15 mM NH_4_^+^. Flasks containing medium with either no NH_4_^+^ or 15 mM NH_4_^+^ were then inoculated using the cultures that had been passaged in replete NH_4_^+^. The inoculation volume was such that NH_4_^+^ carryover was sufficient to sustain a 2-h doubling time for 6 h after inoculation into medium containing no additional NH_4_^+^. Based on inoculation volume, the carryover from inoculation resulted in an initial NH_4_^+^ concentration of less than 2 mM. During the NH_4_^+^ downshift experiment, metabolomics and proteomics analyses were performed at the same time, sampling from the same cultures. The NH_4_^+^ downshift condition had biological triplicates and the NH_4_^+^-replete controls had biological duplicates. The first extraction for metabolomics and proteomics analysis was performed for both conditions 5.5 h after inoculation (OD_600_ of 0.35), when growth was still exponential for both conditions. For the NH_4_^+^ downshift condition, subsequent samples were taken at 1, 1.5, 2, 3, and 6 h after the first sample. For the NH_4_^+^ repletion condition, samples were taken at 1 and 2 h after the first sample.

### NH_4_^+^ upshift.

For the NH_4_^+^ upshift experiment, two rounds of passaging were performed as described for the N_2_ versus NH_4_^+^ experiment, except that all passaging was done in medium containing no NH_4_^+^. Flasks containing medium with no NH_4_^+^ were then inoculated using the cultures that had been passaged under N_2_-fixing conditions. Approximately 6 h after inoculation (OD_600_ of approximately 0.35), the first extraction for metabolomics and proteomics analysis was performed for all cultures. Immediately following this extraction, 1.5 ml of a 1 M NH_4_Cl solution (15 mM final concentration) was added to three experimental cultures, leaving two cultures as N_2_-fixing controls. Subsequently, extractions were taken at 5, 15, 30, 60, and 120 min after addition of NH_4_Cl for the NH_4_^+^ upshift condition. For the N_2_-fixing controls, extractions were taken at 60 and 120 min after the first extraction. This experiment was also performed using ^15^NH_4_Cl, without proteomics sampling. For the ^15^N-labeled iteration, metabolomics time point samples were collected at 1, 5, 15, 30, 60, and 120 min after addition of NH_4_^+^ for the NH_4_^+^ upshift condition and at 15, 30, 60, and 120 min after the first extraction for the N_2_-fixing controls. An additional replicate was also included for the control condition in this iteration, resulting in biological triplicates for both conditions.

### Stable isotope labeling.

[1-^13^C]d-glucose (CLM-420-PK) (98 to 99% isotopic purity), [6-^13^C]d-glucose (CLM-481-PK) (98 to 99% isotopic purity), [1-^2^H]d-glucose (DLM-1150-PK) (98% isotopic purity), [4-^2^H]d-glucose (DLM-9294-PK) (98% isotopic purity), [5-^2^H]d-glucose (DLM-6754-PK) (98% isotopic purity), and ^15^NH_4_Cl (NLM-467-PK) (98 to 99% isotopic purity) were purchased from Cambridge Isotope Laboratories, Inc. For all labeling data in this study, masses were adjusted to account for the natural abundance of ^15^N, ^13^C, or ^2^H using ElemCor (104). For thermodynamics analysis, 10× glucose stock solutions were prepared using autoclaved deionized water and sterilized by passage through a 0.2-μm filter. Both NH_4_^+^ and N_2_ conditions were grown with 15 g/liter glucose to reduce costs. Growth rates were the same as in 20 g/liter glucose. Cultures were passaged as described for the N_2_ versus NH_4_^+^ experiment, except that the final passage before inoculation into experimental flasks was performed in 4-ml volumes in culture tubes containing medium with labeled glucose matching the labeled glucose present in experimental flasks. This was done to minimize unlabeled carryover from inoculation. Metabolite extractions were performed from experimental flasks 5 to 12 h after inoculation, when cultures reached an OD_600_ of 0.35.

### Metabolic flux analysis and goodness of fit.

A Z. mobilis metabolic model was adapted from a report by Jacobson et al. in 2019 and simplified to include only reactions for glucose uptake, ED glycolysis, and ethanol production (85). Reversible reactions were modeled as separate forward and backward reactions. Within the model, cellular H^+^ and CO_2_ were allowed to freely exchange with naturally labeled equivalents. Metabolic flux analysis was performed using the INCA software suite (105), which is implemented in MATLAB and uses the elementary metabolite unit (EMU) framework to simulate isotopic distributions (106). We combined labeling data from our ^13^C and ^2^H tracer experiments ([1-^13^C]glucose, [6-^13^C]glucose, [4-^2^H]glucose, and [5-^2^H]glucose) with glucose uptake and ethanol excretion rates provided by Jake McKinlay (17) to create a single, statistically acceptable flux map using the COMPLETE-MFA technique (107). Glucose tracer inputs were defined by label type (^13^C or ^2^H) and position, but the proportion of unlabeled glucose was allowed to vary by modeling glucose uptake as two glucose inputs, one labeled and one unlabeled, whose relative contribution was controlled by the flux fit optimization process. Final model solutions estimate the unlabeled fraction of glucose at approximately 1%, consistent with nominal tracer purity. Metabolite mass isotopomer distribution (MID) precision was estimated by combining the variance of each measurement across biological replicates and the maximum error observed from naturally labeled metabolites compared to the theoretical MID calculated from natural isotope abundances with a minimum allowable error of 0.3% for each MID, as previously described (85). The combined ^2^H and ^13^C best-fit flux solutions are contained in Table S4 in the supplemental material. Labeling data from ^13^C and ^2^H tracer experiments were used in INCA without prior correction for naturally abundant heavy isotopes.

Intracellular fluxes were estimated by solving a nonlinear least-squares regression problem that minimizes the variance-weighted sum of square residuals (SSR) between simulated and measured isotopic distributions of intracellular and extracellular metabolites. Because the solver does not guarantee a global SSR optimum, we used a random multistart approach until SSR improvement ceased. Using the optimal solution, we calculated 95% confidence intervals for all estimated fluxes by individually varying each flux and testing the sensitivity of the optimal SSR to changes in that flux. Upper and lower bounds were assigned by varying each flux until the SSR was perturbed beyond a critical point corresponding to a chi-square distribution with a single degree of freedom.

A χ^2^ test was used to determine whether estimated fluxes adequately describe the measured labeling data. A correct model and data set have an optimized SSR that falls within a χ^2^ distribution with degrees of freedom equal to the fitted measurements (i.e., non-zero MIDs and measured fluxes, such as uptake and excretion rates) minus the number of independent parameters (i.e., all fluxes estimated by the analysis). We set the critical threshold of our χ^2^ test at 0.05 (95% confidence) and required that optimized SSRs fell within this distribution for acceptance. We attempted to fit labeling data from Z. mobilis grown under NH_4_^+^-replete conditions together with data from N_2_-fixing conditions to a single flux map and were unable to find a statistically acceptable fit, suggesting that these two conditions are distinct metabolic states for Z. mobilis.

### Metabolite extraction.

At the time of extraction, 5 to 10 ml of liquid culture was extracted using a serological pipette. The culture was then rapidly filtered through a 0.45-μm nylon filter (Millipore catalog no. HNWP04700) using a vacuum flask fitted with a sintered glass funnel, separating cells from the growth medium. Immediately after the medium passed through the filter, the cells captured on the filter were plunged into cold extraction solvent, simultaneously quenching metabolism, lysing cells, and dissolving intracellular metabolites. This was done by placing the filter facedown in a small (5.5-cm-diameter) plastic petri dish containing 1.5 ml extraction solvent (40:40:20 methanol-acetonitrile-water; all high-performance liquid chromatography [HPLC] grade). The dish containing extraction solvent was kept on dry ice or an aluminum block that had been stored at −80°C. The entire process of extraction was done in 30 s or less. The filter was then rinsed in the extraction solvent within the dish using a pipette to dislodge remaining cell debris and metabolites. The 1.5 ml of extract was then transferred to a microcentrifuge tube and centrifuged at 16,000 × *g* for 3 min to remove debris. The supernatant was stored at −80°C until analysis by LC-MS. For analysis, 300 to 200 μl of extract was dried down under N_2_ gas. Samples were concentrated three times by resuspension in one-third the dry-down volume of solvent A (see “Metabolomics LC-MS analysis”), vortexed for 10 s, and centrifuged at 16,000 × *g* for 3 min to remove any remaining cell debris. Fifty microliters of the supernatant was then transferred to an HPLC vial for LC-MS analysis.

### Metabolomics LC-MS analysis.

Metabolomics analysis by LC-MS was performed using a Vanquish ultra-high-performance LC (UHPLC) system (Thermo Scientific) coupled to a hybrid quadrupole orbitrap mass spectrometer (Q Exactive, Thermo Scientific), as previously described (23, 48, 108). The chromatography was done using a reverse-phase C_18_ column (1.7-μm particle size, 2.1- by 100-mm column; Acquity UPLC BEH). Solvent A was 97% H_2_O and 3% methanol with 10 mM tributylamine (TBA) and ∼10 mM acetic acid for a pH of 8.2. Solvent B was 100% methanol. The total run time was 25 min. The flow rate was held constant at 0.2 ml/min. The chromatography gradient was as follows: 5% solvent B for 2.5 min, linear increase to 95% B over 14.5 min, maintenance of 95% B for 2.5 min, linear decrease back to 5% B over 0.5 min, maintenance of 5% B for 5 min. Eluent from the column was analyzed by mass spectrometry from the start of the run until 19 min, at which time flow was directed to waste for the remainder of the run. Compounds separated by HPLC were ionized by electrospray ionization (negative polarity) and analyzed by full MS-selected ion monitoring (MS-SIM) with a scanning range of 70 to 1,000 *m/z*, an automatic gain control (AGC) target value of 1 × 10^6^, maximum injection time (IT) of 40 ms, and resolution of 70,000.

### Metabolomics computational analysis.

LC-MS raw files were converted to mzXML format and visualized using MAVEN (109). Peaks were chosen by comparison with retention times obtained using analytical standards. To account for slight signal variation from injection to injection, samples were either run twice (technical duplicate) and averaged or mixed 1:1 with a universally ^13^C-labeled intracellular metabolite sample harvested from E. coli grown in [U-^13^C]d-glucose and normalized by U-^13^C signal, as previously described (48). For each metabolite, signal intensity was divided by OD_600_ to account for variation in culture density between samples. These values were then divided by the average of the three replicates in the control sample to generate fold change values. For N_2_ versus NH_4_^+^, the control sample was NH_4_^+^-replete conditions at early exponential phase. For NH_4_^+^ downshift, the control sample was NH_4_^+^-replete conditions at time zero. For NH_4_^+^ upshift, the control sample was N_2_-fixing conditions at time zero. The log_2_ of the fold change values was then averaged to obtain the data displayed in this study.

### Protein extraction and preparation.

At the time of extraction, 4 ml of culture was collected and cells were pelleted by centrifugation for 3 min at 16,000 × *g*. Supernatant was discarded, and pellets were frozen and stored at −80°C until further analysis. Samples were prepared for LC-tandem MS (LC-MS/MS) analysis by thawing and then lysing cells in 900 μl of methanol, resulting in a final concentration of over 90% methanol. Samples were kept cold at 4°C for 30 min and then centrifuged for 20 min at 15,000 × *g*. Supernatant was removed, and protein extract was air dried at room temperature. The protein pellet was resuspended in 50 μl 8 M urea, 100 mM Tris (pH 8.0)–10 mM TCEP [Tris(2-carboxyethyl)phosphine hydrochloride], and 40 mM chloroacetamide to denature, reduce, and alkylate proteins. Sonication for 10 min ensured that all protein was in solution. The protein concentration was determined with NanoDrop using the A280 method. The protein resuspension was diluted to 1.5 M urea in 100 mM Tris (pH 8.0) and sonicated for 10 min. Trypsin was added at an estimated 50:1 ratio, and samples were incubated overnight (12 h) at ambient temperature. After incubation with digestion enzyme, each sample was prepared for desalting using a 96-well Strata polymeric reversed-phase 10-mg SPE (styrene divinylbenzene) cartridge. Preparation included priming the cartridge wells with 1 ml of ACN (acetonitrile), followed by 1 ml of 0.1% trifluoroacetic acid (TFA). Each sample was acidified with TFA to a final pH of 2.0 or less and then centrifuged for 15 min at 2,000 × *g* to remove all nonprotein material. Acidified sample was then loaded onto the cartridge, washed with 1 ml of 0.1% TFA, and then eluted with 600 μl of 80% ACN–0.1% TFA into a clean 96-well plate to be dried. Samples were resuspended in 0.2% formic acid, and peptide mass was assayed with NanoDrop A280 for a final concentration close to 1 μg/μl.

### Proteomics LC-MS/MS analysis.

Proteomics analysis was performed as previously described (22, 25, 26). For each analysis, 1 μg of peptides was loaded onto a 75-μm-inside-diameter (i.d.), 30-cm-long capillary with an embedded electrospray emitter and packed in a 1.7-μm-particle-size C_18_ BEH column (stationary phase). The mobile phases used were as follows: phase A, 0.2% formic acid; phase B, 0.2% formic acid–70% acetonitrile. The peptides were eluted with a gradient of acetonitrile increasing from 0% to 50% B over 74 min followed by a 1-min increase to 100% B, 5 min sustained at 100% B, and a final 10 min of equilibration in 100% A. The eluting peptides were analyzed with an Orbitrap Eclipse (Thermo Fischer Scientific) mass spectrometer. Survey scans were performed at a resolution of 240,000 with an isolation analysis at *m/z* 300 to 1,350 and 250% normalized automatic gain control (AGC) target. Data-dependent top-speed (1-s) MS/MS sampling of peptide precursors was enabled with dynamic exclusion set to 10 s on precursors with charge states 2 to 5. Data-dependent MS/MS sampling was performed with 0.5-Da quadrupole isolation, with fragmentation by higher-energy collisional dissociation (HCD) with a normal collisional energy (NCE) value of 300%. The mass analysis was performed in the ion trap using the “turbo” scan speed for a mass range of 150 to 1,350 *m/z* with a maximum inject time of 14 ms, and the normalized AGC target set to 300%.

### Proteomics computational analysis.

Raw files were analyzed using MaxQuant 1.5.8.3 (110). Spectra were searched using the Andromeda search engine against a decoy target list. Label-free quantitation and match between runs were toggled on, MS/MS tolerance was set to 0.4 Da, and the number of measurements for each protein was set to 1. Default values were used for all other analysis parameters. The peptides were grouped into subsumable protein groups and filtered to reach 1% FDR, based on the target decoy approach. The fasta database “Zm4.CDS.AA.fasta” was used to generate the protein list utilizing the ZM4 tag for protein names. Using RStudio, the label-free quantitation (LFQ) values and protein intensities in each sample were log_2_ transformed and filtered to contain proteins that fulfill a >50% cutoff of measurements across the samples. The average and standard deviation were calculated across the replicates for each protein, and fold changes are relative to control time zero for each experiment.

### Statistical analysis.

Statistical analysis for metabolomics and proteomics data sets was performed in R. For metabolomics, raw signal intensity was normalized by OD_600_ and then log_2_ transformed before statistical analysis. For proteomics, the log_2_ of LFQ values was used. For both metabolomics and proteomics time courses, a repeated-measures analysis of variance (ANOVA) test was performed for each metabolite or protein, comparing time points within the treatment group. This tests the null hypothesis that the mean value for metabolite or protein abundance was not different at any time during the time course. The *P* values from the repeated-measures ANOVA tests were then adjusted for multiple hypothesis testing using the Benjamini-Hochberg method to control for false discovery rate (FDR) (111). In this case, an FDR-adjusted *P* value below 0.05 indicates that less than 5% of proteins or metabolites identified as changing during changes in NH_4_^+^ availability are false positives. For metabolomics during continuous N_2_-fixing conditions compared to continuous NH_4_^+^-replete conditions, a two-way repeated-measures ANOVA test was performed to test the null hypothesis that the average metabolite abundance was not different between N_2_-fixing and NH_4_^+^-replete conditions, independent of growth stage. The *P* values from this test were adjusted for multiple hypothesis testing using the Benjamini-Hochberg method. For proteomics during continuous NH_4_^+^ availability, an unpaired *t* test was performed to test the null hypothesis that the average protein abundance was not different between N_2_-fixing and NH_4_^+^-replete conditions. The *P* values from this test were adjusted for multiple hypothesis testing using the Benjamini-Hochberg method. For ^15^N isotope tracers, a repeated-measures ANOVA was performed to test the null hypothesis that the average fraction of M + 2 glutamine was not different at any time point between 15 min and 2 h. For thermodynamic analysis using isotopic labeling, statistical analysis was performed in GraphPad Prism. For this data set, an unpaired *t* test was performed for each metabolite shown in Fig. 12, testing the null hypothesis that the average fraction of reverse-flux-associated labeled form was not different between N_2_-fixing and NH_4_^+^-replete conditions.

### Data availability.

The mass spectrometry proteomics data have been deposited in the ProteomeXchange Consortium via the PRIDE (112) partner repository with the data set identifier PXD028526. Metabolomics data have been deposited in the Open Science Framework (https://osf.io) (113) under the project “N2 fixation in Zymomonas mobilis” at https://doi.org/10.17605/OSF.IO/GJVYW.
